# Design and Tunability of Thermochromic Polymers Based on Dye–Developer–Solvent Systems: Toward Non-Toxic Materials

**DOI:** 10.1021/acsomega.6c06914

**Published:** 2026-10-07

**Authors:** Chan Qi-Hua, Zuratul Ain Abdul Hamid, Ku Marsilla Ku Ishak, Yazmin Bustami

**Affiliations:** † School of Materials and Mineral Resources Engineering, 65270 Universiti Sains Malaysia Engineering Campus, Nibong Tebal, Pinang 14300, Malaysia; ‡ School of Biological Sciences, Minden Heights, 26689 Universiti Sains Malaysia, Gelugor, Pulau Pinang 11800, Malaysia

## Abstract

Thermochromic polymers have had significant development over the years through consistent research. Specifically, polymers that involve an embedded ternary thermochromic compound, which consists of a dye, color developer, and cosolvent, have garnered a lot of attention for the tunability of their color-changing properties. These compounds exhibit color changes through the mechanism of proton transfer between the dye and developer species, and the presence of a solvent is crucial to facilitating this phenomenon. By manipulating the type of each component within the dye-developer-solvent (DDS) compound, the color, direction of color change, and phase transition temperature can be adjusted. The fabrication of thermochromic polymers with DDS compounds can be done through encapsulation, impregnation into a porous material, coating, or a combination of encapsulation and compounding with a polymer matrix. Potential applications of temperature-sensitive polymers include temperature sensors and heat regulators in packaging, clothing, medical devices, and architecture. Research efforts are being continuously made on DDS compounds to make them safer and more eco-friendly with the replacement of conventional components with biobased materials. This review provides a comprehensive overview of the working principle of ternary thermochromic DDS compounds, the tunability of the thermochromic properties, the current progress of research on utilizing DDS compounds in polymers, and the prospects in developing nontoxic thermochromic polymers for food packaging and medical applications.

## Graphical abstract

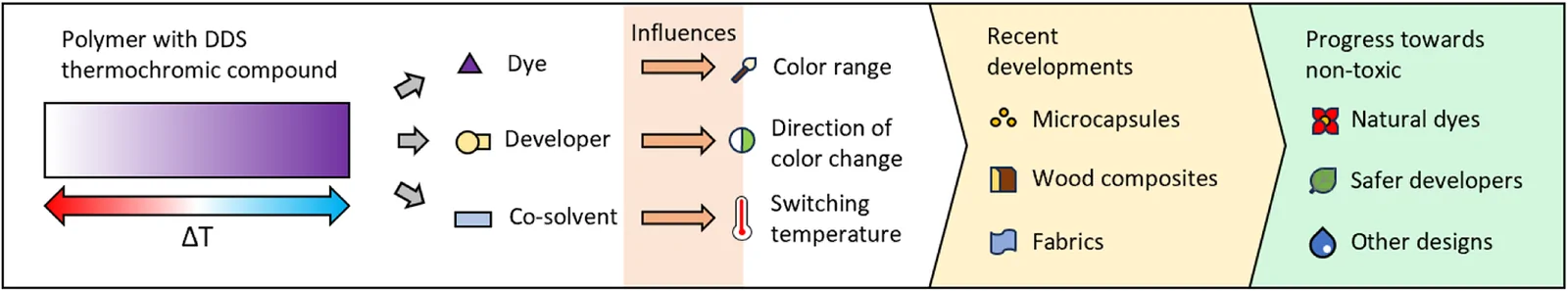

## 1 Introduction

Thermochromic polymers have emerged as an important class of functional materials capable of converting temperature variations into visible optical signals, enabling applications in smart packaging, wearable systems, medical diagnostics, and energy-efficient building materials. Thermochromic polymers are defined as phase-change materials that undergo a transition from one color to another with a change in temperature. Unlike conventional temperature-sensing technologies, thermochromic systems offer a rapid, passive, and visual indication of thermal changes without the need for external instrumentation, making them attractive for real-time monitoring applications. Thermochromic polymers can be broadly classified into three categories: (1) inherently thermochromic polymers, (2) polymers embedded with thermochromic compounds, and (3) polymers embedded with nonthermochromic additives.^1,2^ The difference between thermochromic compounds and nonthermochromic additives is that the former has the ability of changing colors in response to temperature, while the latter relies on temperature-sensitive structural changes in the matrix to stimulate color changes in the dye.

Thermochromic compounds themselves may be organic, inorganic, hybrid systems, or intrinsically thermochromic polymers.^3^ This Review focuses specifically on organic DDS systems incorporated into polymer matrices. Among the various thermochromic systems developed to date, the most common type of thermochromic pigment on the market is leuco dye-based.^4^ Leuco dyes are a class of synthetic dyes consisting of fluoran, diphenylmethane, phthalide, spiropyran, spirolactone, fulgide, and polymethine dyes.^5–7^ The Greek root word *leukos* means white, describing the ability of the dye to change between colored and colorless states.^8^ To form a thermochromic DDS compound, the color formers are generally mixed with two other components, namely, the color developer and cosolvent. The dye and developer serve as the electron donor and acceptor, respectively, and both form a complex that changes the color of the dye.^9^ Changes in the cosolvent between solid and liquid forms affect the developer-solvent interaction. When the developer-solvent interaction is strong, the dye-developer complex is broken.^10^ This alters the state of the dye and a macroscopic color change of the compound is observed.

The versatility of DDS systems lies in their high tunability, as the selection and composition of each component directly influence key performance parameters such as color intensity, switching temperature, and the direction of thermochromism. First, the color of the dye depends on its molecular structure, which determines its interaction with light, as absorbance at different wavelengths within the visible light spectrum gives a different product of light, which is seen as a specific color.^11^ Second, depending on the similarity in length of the hydrocarbon chain between the developer and cosolvent, the DDS compound can either change from colored to colorless, or vice versa, upon heating.^12,13^ Third, the melting point of the cosolvent determines the temperature range at which color changes occur.^14^

Despite significant progress, several critical challenges remain in translating DDS-based thermochromic polymers into advanced engineering applications. First, while tunability has been widely demonstrated, the lack of a systematic understanding of structure–property relationships limits the rational design of DDS systems with targeted performance. The interplay between developer–dye interactions, solvent crystallization behavior, and polymer matrix compatibility remains insufficiently consolidated into practical design guidelines. Second, many conventional DDS systems rely on synthetic components such as bisphenol A (BPA), raising concerns regarding toxicity, migration, and long-term safety, particularly for applications in food packaging and biomedical devices.^15,16^ Although emerging studies have explored biobased dyes and alternative developers, the development of nontoxic thermochromic systems remains fragmented and lacks a unified framework.

In addition, fabrication strategies such as microencapsulation, impregnation, and direct blending introduce further complexity, as they influence thermal stability, response kinetics, and durability of the thermochromic response. A comprehensive evaluation of these processing–structure–property relationships is therefore essential to bridge the gap between laboratory-scale demonstrations and practical material design.

This Review aims to address these challenges by providing a critical and structured analysis of thermochromic polymers based on DDS systems. Specifically, the review (i) elucidates the fundamental mechanisms governing thermochromic behavior, (ii) establishes correlations between material composition and tunable properties, (iii) evaluates recent developments in polymer-integrated DDS systems with emphasis on processing strategies, and (iv) highlights emerging approaches toward nontoxic and sustainable thermochromic materials. By integration of these aspects, this work seeks to provide design-oriented insights that can guide the development of next-generation thermochromic polymers for advanced functional applications.

## 2 Principle of Thermochromic Color Change

Thermochromic behavior in DDS systems is governed by reversible intermolecular interactions among a leuco dye, a color developer, and a phase-change solvent. Typically, the dye functions as an electron donor, while the developer acts as a proton donor or electron acceptor. The solvent, commonly a long-chain organic compound such as a fatty alcohol or fatty acid, regulates these interactions through its phase transition. At temperatures below the phase transition temperature, the solvent exists in a crystalline solid state, which promotes close proximity between the dye and developer. Under these conditions, strong dye–developer interactions induce proton transfer, leading to structural transformation of the dye into its ring-opened form, as illustrated in Figure 1a. This conjugated structure absorbs visible light, resulting in a colored state.

**Figure 1:**
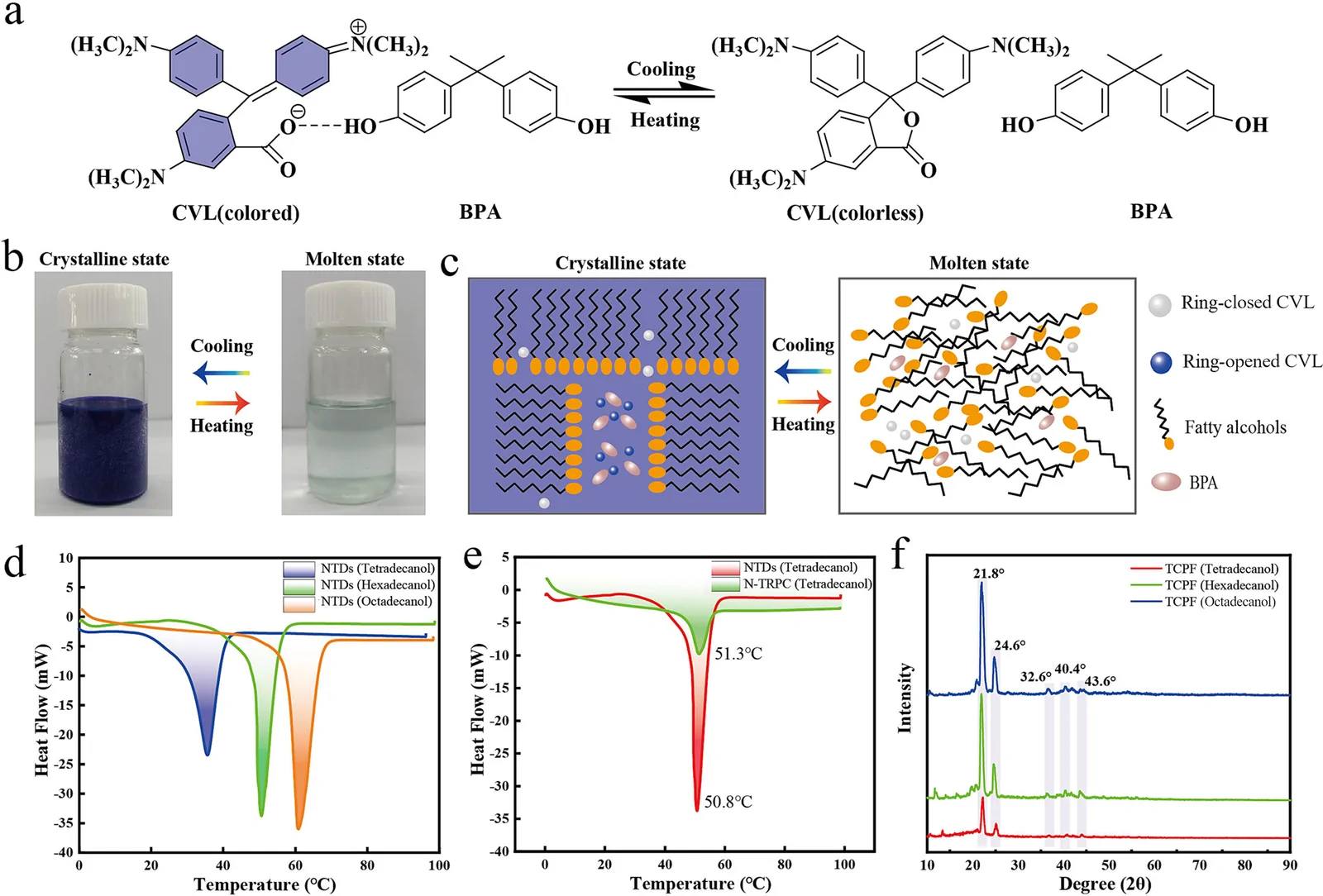
a) Color development principle of negative thermochromic dyes. b) The optical photographs illustrating the thermal discoloration behavior of negative thermochromic dyes. c) The thermochromic principle of negative thermochromic dyes. d) DSC curves of negative thermochromic dyes; the three curves correspond to different fatty alcohols: *n-*tetradecanol, *n-*hexadecanol and *n-*octadecanol, respectively. e) DSC curves of negative thermochromic dyes and thermally responsive photonic crystals. f) XRD spectra of thermochromic polymer films in the crystalline state; the three curves correspond to different fatty alcohols: *n-*tetradecanol, *n-*hexadecanol and *n-*octadecanol, respectively.^13^ (“Photograph courtesy of ‘Tan et al.’,Copyright 2023).”).

Upon heating above the solvent melting temperature, the solvent transitions into a liquid phase, weakening the dye–developer interaction by increasing molecular mobility and promoting solvent–developer association. As a result, the protonated dye reverts to its ring-closed form, disrupting conjugation and causing the material to become colorless. The overall thermochromic process, including the reversible transition between colored and colorless states during heating and cooling, is schematically presented in Figure 1c. This process is reversible, as cooling restores the crystalline solvent structure, reestablishing dye–developer interactions and recovering the colored state. The thermally driven equilibrium between dye–developer and developer–solvent interactions underpins the thermochromic response and determines key functional characteristics such as color intensity, switching temperature, and reversibility. Depending on the relative strength of these interactions, DDS systems may exhibit negative thermochromism (color fading upon heating) or positive thermochromism (color formation upon heating). Understanding this interaction-driven mechanism provides the foundation for the rational design of DDS systems with tailored switching temperatures and optical responses.

## 3 Tunability of Thermochromic Properties

The versatility of the DDS thermochromic systems arises from the ability to tailor their optical and thermal responses through compositional design. The key tunable parameters include the color range, direction of color change, and switching temperature, all of which are governed by intermolecular interactions and phase transition behavior. Understanding these relationships enables the rational design of thermochromic polymers for specific applications.

### 3.1 Color Range

The color that a thermochromic compound exhibits at different temperatures is controlled by the color range of the dye at different states. Common pH-sensitive leuco dyes used in DDS systems and their corresponding color transitions are summarized in Table 1. These dyes typically exhibit reversible transitions between colored (ring-opened) and colorless (ring-closed) states, enabling clear visual responses to temperature changes. Fluoran-based dyes, such as crystal violet lactone (CVL) and 6′-(diethylamino)-1′,3′-dimethylfluoran (DDF), are the most widely used due to their high color intensity and sharp contrast between colored and colorless states. In contrast, azo dyes such as methyl red generally exhibit lower color contrast and may require more precise control of environmental conditions to achieve distinct transitions. Additionally, most dyes operate within similar pH-responsive mechanisms, indicating that the primary differentiation lies in their optical properties rather than fundamental switching behavior. This suggests that dye selection is largely governed by application-specific requirements such as visibility, sensitivity, and desired color output.^17^

**Table 1:** List of Dyes and Their Colors at Different pH Conditions

		Color changes			
Colorant	Systematic name	Acidic	Neutral	Alkaline	Reference
BCP	5′,5″-dibromo-o-cresolsulfophthalein	Yellow	Yellow	Purple	^26,27^
BP	3,3′-bis(1-n-octyl-2-methylindol-3-yl)phthalide	Red	Colorless	Colorless	^28,29^
CVL	6-(dimethylamino)-3,3-bis[4-(dimethylamino)phenyl]-2-benzofuran-1-one	Violet	Colorless	Colorless	^9,25^
DDF	6′-(Diethylamino)-1′,3′-dimethylfluoran	Red	Colorless	Colorless	^30^
Methyl red	2-(N,N-dimethyl-4-aminophenyl) azobenzenecarboxylic acid	Red	Colorless	Colorless	^31^
SO	2′-(dibenzylamino)–6′-(diethylamino)spiro[isobenzofuran-1(3H),9′-[9H]xanthene]–3-one	Blue/purple	Colorless	Colorless	^28,29^

Recent studies have explored the use of multiple thermochromic dyes or pigments to achieve multistage color transitions across different temperature ranges. For example, systems incorporating dyes with distinct activation temperatures can produce sequential color changes, enabling more precise temperature indication. However, such approaches remain largely empirical, as the interaction between different dyes within a DDS system is not fully understood. Challenges such as overlapping color responses, reduced contrast, and interference between dye–developer equilibria can limit the effectiveness of multidye systems. Consequently, systematic design strategies for achieving predictable and well-resolved multicolor thermochromic responses are still lacking.

### 3.2 Direction of Color Change

The compatibility of the developer with the cosolvent determines the direction of color change, which is governed by the length of the hydrocarbon chains in the developer.^12^ In negative thermochromic compounds, the developer for example, BPA has a short hydrocarbon chain. Due to the difference between the developer and the fatty cosolvent, the developer tends to form better interactions with the dye than the cosolvent in the solid state. Hence, the compound appears colored below phase transition temperature, as shown in Figure 2. As heat is supplied to the compound, the cosolvent melts and disrupts the dye–developer interaction, and the compound becomes discolored upon heating. This phenomenon is reversed for the positive thermochromic compounds.

**Figure 2:**
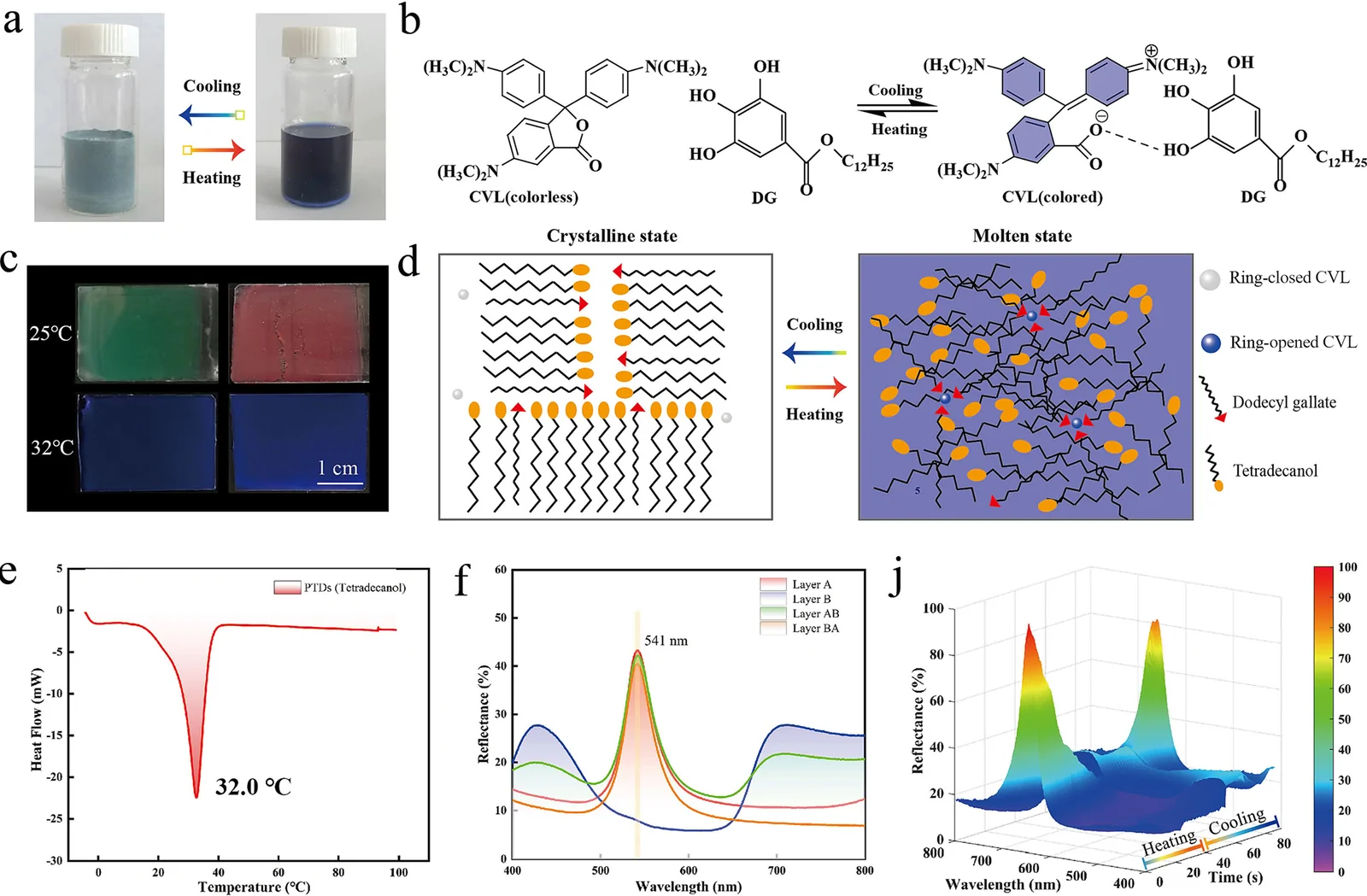
a) The optical photographs of positive thermochromic dyes. b) Color development principle of positive thermochromic dyes. c) The thermochromic principle of positive thermochromic dyes. d) The optical photographs illustrating the thermal discoloration behavior and the thermochromic principle of positive thermochromic dyes. e) Optical photographs of positive thermally responsive photonic crystal films at 25 and 50 °C. f) Reflectance spectra of Layer A, Layer B, Layer AB, and Layer BA of positive thermally responsive photonic crystal films. j) Dynamic reflectance curves of positive thermally responsive photonic crystal films.^13^ (“Photograph courtesy of ‘Tan et al.’,Copyright 2023).”).

As illustrated in Figure 1a–c, developers with shorter hydrocarbon chains (e.g., BPA) exhibit lower compatibility with long-chain solvents, favoring dye–developer interaction in the solid state and resulting in negative thermochromism (colored at low temperature, colorless upon heating). In contrast, developers with longer hydrocarbon chains (e.g., alkyl gallates) show higher affinity toward the solvent, promoting developer–solvent interaction in the solid state and leading to positive thermochromism (colorless at low temperature, colored upon heating),^18^ as depicted in Figure 2a, 2b and 2d.

However, the transition between positive and negative thermochromism is not solely dictated by chain length but also depends on factors such as polarity, hydrogen-bonding capability, and the degree of phase separation between components.^19^ For instance, highly compatible developer–solvent systems may suppress dye–developer interactions even at low temperatures, resulting in weaker color intensity or delayed response. Conversely, strong dye–developer interactions can lead to sharper transitions but may reduce reversibility due to stronger intermolecular binding and a lower tendency for phase separation.^20^

These observations highlight that the direction of thermochromism arises from a dynamic balance between competing intermolecular interactions rather than a single structural parameter. Achieving an optimal balance between dye–developer and developer–solvent interactions is critical for ensuring both clear color contrast and reversible thermochromic behavior.

### 3.3 Switching Temperature

The main factor that controls the phase transition temperature of a thermochromic DDS compound is the melting point of the cosolvent. The thermochromic transition is triggered by the solid–liquid phase change of this component. Common phase-change solvents include fatty acids, fatty alcohols, alkanes, and esters, which span a wide range of melting temperatures, as summarized in Table 2. A comparison of the solvents listed in Table 2 reveals that long-chain hydrocarbons exhibit a systematic increase in melting temperature with chain length,^21^ enabling coarse tuning of switching temperature through solvent selection. However, this approach is limited by the discrete melting points of pure compounds, restricting precise control for targeted applications.

**Table 2:** Phase-Change Materials Used in Literature

Type	Solvent (alternative name)	Melting point [°C]	Latent heat of fusion [J g^–1^]	Reference
Alkane	*n*-tetradecane	5	261	^25^
	*n*-octadecane	28	243	^32^
Fatty acid	*n*-decanoic acid (capric acid)	30–32	145	^33–35^
	*n*-dodecanoic acid (lauric acid)	43–45; 51	176–194	^22,24,34,35^
	*n*-tetradecanoic acid (myristic acid)	53–56; 63	186–198	^22,24,32–34^
	*n*-hexadecanoic acid (palmitic acid)	62–66; 69	205–214; 240	^22,24,32,34,36^
	*n*-octadecanoic acid (stearic acid)	67–69; 78	211–244	^22,24,34,37^
Fatty alcohol	*n*-dodecanol (lauryl alcohol)	24–27	158–218	^21,25,29^
	*n*-tetradecanol (myristyl alcohol)	35–45	187–237	^12,21,29,30,38–41^
	*n*-hexadecanol (palmityl alcohol)	49–52; 57–59	219–245	^21,22,29,30,42,43^
	*n*-octadecanol (stearyl alcohol)	58–62	211–279	^12,29,34,40,44,45^
	*n*-eicosanol (arachidyl alcohol)	65	284	^29^
Ester	Dodecyl decanoate	32	199	^46^
	Tetradecyl tetradecanoate	37.4	-	^43^

To achieve finer control, binary eutectic mixtures are widely employed. As shown in Table 3, combining two phase-change materials can significantly depress the melting temperature relative to individual components. The phase transition behavior of such mixtures can be estimated using the Schrader equation:

1 $$T_{ab}={[\frac{1}{T_{i}}-\frac{R\ln X_{i}}{\Delta H_{i}}]}^{-1}\left(i=a,b\right)$$

where *a* or *b* is one of the binary eutectic mixture components, *T*_*ab*_ is the phase transition temperature of the mixture, *T*_*i*_ is the phase transition temperature of *i*, Δ*H*_*i*_ is the latent heat of *i*, *X*_*i*_ is the molar fraction of *i*, and *R* is the gas constant, which equals to 8.314 J mol^–1^ K^–1^.^22^

**Table 3:** Properties of Binary Eutectic Mixtures from Literature^a^

Type	Binary mixture	Mass ratio	Eutectic point [°C]	Latent heat of fusion [J g^–1^]	ref.
Acid/acid	*n*-decanoic acid/*n*-dodecanoic acid	60.5:39.5	17.7	155.2	^23^
	*n*-decanoic acid/*n*-dodecanoic acid	67.0:33.0	21.0	-	^35^
	*n*-decanoic acid/*n-*tetradecanoic acid	72.0:28.0	19.4	150.9	^23^
	*n*-decanoic acid/*n-*tetradecanoic acid	78.0:22.0	20.5	153.0	^33^
	*n*-decanoic acid/*n-*hexadecanoic acid	82.8:17.2	22.1	165.6	^23^
	*n*-decanoic acid/*n-*octadecanoic acid	88.3:11.7	23.4	150.5	^23^
	*n*-dodecanoic acid/*n-*tetradecanoic acid	60.0:40.0	36.1	158.9	^23^
	*n*-dodecanoic acid/*n-*hexadecanoic acid	69.8:30.2	37.8	164.0	^23^
	*n*-dodecanoic acid/*n-*octadecanoic acid	80.5:19.5	39.9	167.1	^23^
	*n*-tetradecanoic acid/*n-*hexadecanoic acid	60.8:39.2	44.1	182.2	^23^
	*n*-tetradecanoic acid/*n-*hexadecenoic acid	-	46.5	196.4	^28^
	*n*-tetradecanoic acid/*n-*octadecanoic acid	71.5:28.5	44.8	186.9	^23^
	*n*-tetradecanoic acid/*n-*octadecanoic acid	-	46.5	203.8	^28^
	*n*-hexadecanoic acid/*n-*octadecanoic acid	61.0:39.0	56.0	181.0	^24^
	*n*-hexadecanoic acid/*n-*octadecanoic acid	63.5:36.5	57.1	193.0	^23^
	*n*-hexadecanoic acid/*n-*octadecanoic acid	-	58.4	239.4	^28^
Acid/alcohol	*n*-dodecanoic acid/*n-*hexadecanol	60.2:39.8	47.6	200.0	^22^
	*n*-dodecanoic acid/*n-*hexadecanol	53.0:47.0	32.9	230.8	^47^
	*n*-hexadecanol/*n-*tetradecanoic acid	54.0:46.0	50.9	210.0	^22^
	*n*-hexadecanol/*n-*hexadecanoic acid	63.4:36.6	55.5	220.0	^22^
	*n*-hexadecanol/*n-*octadecanoic acid	77.1:22.9	56.9	220.0	^22^
Acid/alkane	*n*-octadecane/*n-*tetradecanoic acid	89.7:10.3	26.5	236.0	^32^
	*n*-octadecane/*n-*hexadecanoic acid	95.0:5.0	27.2	238.0	^32^
Alcohol/alcohol	*n*-dodecanol/*n-*tetradecanol	74.4:25.6	13.5	-	^21^
	*n*-dodecanol/*n-*tetradecanol	73.0:27.0	–2.2; 21.6	143.8	^29^
	*n*-dodecanol/*n-*hexadecanol	87.4:12.6	19.4	-	^21^
	*n*-dodecanol/*n-*hexadecanol	85.7:14.3	18.3	155.4	^29^
	*n*-dodecanol/*n-*octadecanol	91.5:8.5	21.9	-	^21^
	*n*-dodecanol/*n-*octadecanol	95.1:4.9	19.3	202.0	^29^
	*n*-dodecanol/*n-*eicosanol	97.9:2.1	20.7	218.2	^29^
	*n*-tetradecanol/*n-*hexadecanol	68.4:31.6	32.1	-	^21^
	*n*-tetradecanol/hexadecanol	67.0:33.0	2.2; 40.1	185.5	^29^
	*n*-tetradecanol/*n-*octadecanol	86.0:14.0	–1.8; 39.2	219.9	^29^
	*n*-tetradecanol/*n-*eicosanol	93.2:6.8	35.9	227.3	^29^
	*n*-hexadecanol/*n-*octadecanol	63.5:36.5	45.7	-	^21^
	*n*-hexadecanol/*n-*octadecanol	67.9:32.1	9.4; 51.0	201.8	^29^
	*n*-hexadecanol/*n-*eicosanol	79.3:29.7	46.6	204.8	^29^
	*n*-octadecanol/*n-*eicosanol	63.8:36.2	34.0; 58.5	223.2	^29^
Alcohol/alkane	*n*-dodecanol/*n*-tetradecane (with CVL/BPA)	10.0:90.0	7.2	238.5	^25^

a Some samples recorded two melting peaks. Other components of mixtures used in thermochromic compounds are specified in brackets.

By varying the molar fraction, researchers can generate a series of predicted melting temperatures for each component and construct phase diagrams. The intersection of these curves corresponds to the eutectic composition, where the mixture exhibits a single minimum melting temperature. Several studies have applied this approach to design thermochromic systems with tailored switching temperatures, demonstrating that small adjustments in the molar ratio can result in significant shifts in the phase transition temperature.^21^ In experimental studies, the melting temperature of binary mixtures is typically determined by using differential scanning calorimetry (DSC), which provides both thermal transition temperatures and latent heat values. By preparing mixtures with varying molar fractions and recording their melting behavior, we can construct phase diagrams to identify eutectic compositions and corresponding minimum melting temperatures. While theoretical predictions based on thermodynamic models offer useful guidance, discrepancies between calculated and experimental values are commonly observed due to nonideal mixing, supercooling effects, and interactions with the polymer matrix or encapsulation environment. Therefore, experimental validation using thermal analysis remains essential for accurately determining the switching temperature of DDS systems.

### 3.4 Energy Storage

Apart from phase transition temperature, compounds with long-chain alkyl solvents and eutectic mixtures of those have the ability to store and release latent heat during thermal cycles. Tables 2 and 3 list the latent heat of cosolvents and the mixtures used in the literature. As the alkyl chain length and molecular weight of fatty acids and alcohols increase, intermolecular van der Waals forces increase in strength, causing the latent heat to increase. Similar to the phase transition temperature, the latent heat of fusion can be tuned by producing binary eutectic mixtures and manipulating the ratio of each component. The theoretical latent heat of fusion of a mixture can be calculated with eq 2.^23^

2 $$\Delta H_{ab}=T_{ab}\overset{n}{\underset{i=1}{\sum }}\left(\frac{X_{i}\Delta H_{mi}}{T_{mi}}\right)\left(i=a,b\right)$$

Individual and binary eutectic mixtures of solvents with long alkyl chains can be used for latent energy storage applications at low to moderate temperature ranges.^24^ This can be useful in applications that require the temperature of a certain product to be kept constant, such as maintaining a cool temperature in vaccine storage.^25^

## 4 Recent Development of Thermochromic DDS Compounds in Polymers

Recent developments in thermochromic polymers have largely focused on integrating DDS systems into polymer matrices to enhance processability, stability, and functionality. These approaches can be broadly categorized as microencapsulation, direct incorporation into porous or solid matrices, and surface coating strategies. While all methods aim to preserve thermochromic functionality, they introduce distinct effects on the thermal behavior, color intensity, durability, and response kinetics.

A comparison of the reported systems (summarized in Table 4) reveals that the performance of thermochromic polymers is governed not only by DDS composition but also by the method of incorporation and the resulting microstructure. In particular, confinement effects, interfacial interactions, and dispersion quality play critical roles in determining thermochromic performance.

**Table 4:** Thermochromic DDS Compounds Prepared in Literature

Thermochromic compound	Polymeric material/matrix	Thermochromic properties	Proposed application	Reference
BDF/BPA/*n-*tetradecanol	MUF microcapsules; PVA/cotton fabric	Deep red (25 °C) →Colorless (39 °C)	High-performance thermochromic textile	^19^
BDF/DG/*n-*tetradecanol	2-hydroxy-2-methyl-1-phenyl-1-propanone/poly(ethylene glycol) phenyl ether acrylate/linen fabric	Pale white (40 °C) →Deep red (59 °C)	High-performance thermochromic textile	^19^
BP/BPA/binary fatty alcohol eutectic mixture	Delignified eucalyptus fiber skeleton aerogel	Dark red (25 °C) →White (32 °C)	Home automation, passive infrastructural energy-saving, luminescent response switches	^29^
BP/BPA/binary fatty alcohol eutectic mixture	Delignified eucalyptus fiber skeleton coated with PVA/PG	Light red (<38 °C) →Dark red (>38 °C)	Building energy saving, intelligent windows, decoration	^28^
CVL/2-naphthol/methyl dodecanoate	Methylcellulose microcapsules	Blue (−5 °C) →White (0 °C)	Cold chains, fabrics	^65^
CVL/BPA/*n-*decanoic acid	MUF microcapsules	Blue (20 °C) →Light blue (60 °C)	Photothermal conversion material	^51^
CVL/BPA/cresol red/*n-*hexadecanol	-	Gray (25 °C) →Red (48 °C)	Anticounterfeiting and sensors	^73^
CVL/BPA/1-dodecanol	PLA/surface-modified cellulose nanocrystal nanofibers	Dark blue (0.5 °C) → White (20 °C)	Temperature sensors	^67^
CVL/BPA/dodecyl decanoate	MF microcapsules	Blue (20 °C) →Yellow (35 °C)	Intelligent textiles	^46^
CVL/BPA/ethyl stearate or butyl stearate	Filter paper	Blue → White	Thermal storage, information storage, smart temperature sensor	^9^
CVL/BPA/*n-*hexadecanol	MF microcapsules	Blue (25 °C) → White (65 °C)	Smart fabric, commodity packaging, thermal sensors, coatings, solar energy storage	^42^
CVL/BPA/*n-*hexadecanol	Silica microcapsules	Blue (25 °C) → White (60 °C)	Smart textiles, smart coatings, luminescent thermal sensors, anticounterfeiting	^50^
CVL/BPA/*n-*nonanoic acid/isopropanol	MUF microcapsules with gallic acid and/or nano-TiO_2_	Dark blue (0 °C) → Light blue (30 °C)	Visual thermal management	^56^
CVL/BPA/*n-*octadecanol	Sodium alginate/hydroxypropyl methylcellulose/polyvinyl butyral/ ethyl cellulose coated cardboard	Dark blue (30 °C) → Colorless (55 °C)	Food packaging	^68^
CVL/BPA/*n-*octadecanol	PMMA microcapsules/silicone electronic potting materials	Blue (20 °C) → White (60 °C)	Thermal management and monitoring of lithium batteries and integrated electronics	^55^
CVL/BPA/phase change wax	Silica microcapsules; silicone rubber matrix	Dark blue (25 °C) → White (40 °C)	Coatings, anticounterfeiting, wearable electronics	^66^
CVL/BPA/1-tetradecanol	Methylated MF microcapsules	Blue (25 °C) → White (45 °C)	Protective clothing	^38^
CVL/BPA/*n-*tetradecanol	PVA-WPU membrane	Light blue (15 °C) → White (40 °C)	Wound dressing	^71^
CVL/BPA/*n-*tetradecanol	Cyclohexane coating onto polystyrene photonic crystal opal	Blue (25 °C) → Colorless (50 °C)	Anticounterfeiting, steganography, smart windows	^13^
CVL/BPA/*n-*tetradecanol	Diatomaceous earth composite; tannic acid-modified bamboo powder/high-density polyethylene	Dark blue (25 °C) → Gray-white (35 °C)	Construction and furniture	^74^
CVL/BPA/*n-*tetradecanol	MUF microcapsules; PET fabric with humidity-sensitive discoloration materials (thymol blue/cresol red)	Green (30 °C) → Yellow (40 °C) → Orange (50 °C) → Red (80 °C)	Textile fashion, environmental monitoring, healthcare, storage humidity sensor, information encryption	^60^
CVL/BPA/1-tetradecanol	Poplar wood/nano-CaCO_3_	Dark blue (<38 °C) → Off-white (>38 °C)	Information storage, decoration, furniture, energy-efficient building solutions	^58^
CVL/BPA/*n-*tetradecanol	Sodium alginate/CaCl_2_ microcapsules	Blue purple (26 °C) → White (50 °C)	Solar thermal utilization	^52^
CVL/BPA/*n-*tetradecanol/*n-*hexadecanol	MF microcapsules; PDMS printing paste; cotton fabrics	Blue (20 °C) → Colorless (40 °C)	Temperature-visualization sensors, unidirectional moisture-wicking coatings, thermoregulatory fabric	^63^
CVL/BPA/1-tetradecanol/1-octadecanol	MF microcapsules; graphene-coated fabric	Blue (20 °C) → White (37 °C)	Personal thermal management, cooling of electronic devices	^40^
CVL/BPA/ *n-*tetradecane/*n-*dodecanol	MF microcapsules	Blue-purple (2 °C) → White (8 °C)	Pharmaceutical cold storage	^25^
CVL/BPAF/*n-*butyl stearate	Silane-terminated polyurethane microcapsules	Blue (−10 °C) → White (40 °C)	Wearable electronic devices, smart material coatings, temperature-sensitive medical systems	^53^
CVL/BPAF/tetradecyl benzoate	MF microcapsules	Blue (−20 °C) → Light blue (20 °C)	Energy storage, building energy efficiency, anticounterfeiting	^54^
CVL/BPAF/hexadecyl benzoate	MF microcapsules	Blue (0 °C) → Light blue (40 °C)	Energy storage, building energy efficiency, anticounterfeiting	^54^
CVL/BPAF/octadecyl benzoate	MF microcapsules	Blue (−10 °C) → Light blue (40 °C)	Energy storage, building energy efficiency, anticounterfeiting	^54^
CVL/DG/methyl dodecanoate	Methylcellulose microcapsules	Blue (0 °C)→ White (10 °C)	Cold food packaging	^48^
CVL/DG/*n-*tetradecanol	Cyclohexane coating onto polystyrene photonic crystal opal	Light blue (25 °C) →Dark blue (32 °C)	Anticounterfeiting, steganography, smart windows	^13^
CVL/ethyl octanoate/methyl octanoate	MF microcapsules; PVA coating; delignified hinoki wood	Blue-purple (20 °C) → Light yellow (31 °C)	Home décor, temperature sensors, anticounterfeit labeling	^57^
CVL/phenolphthalein/1-tetradecanol	PMMA microcapsules; cotton fabric	Blue (20 °C) → Colorless (50 °C)	Smart textiles, aerospace engineering, wearable electric systems	^61^
DDF/2-naphthol/methyl dodecanoate	Methylcellulose microcapsules	Red (−5 °C)→ White (0 °C)	Cold chains, fabrics	^65^
DDF/CVL/BPA/1-tetradecanol/1-hexadecanol	MF microcapsules; fabric	Purple (20 °C) → Blue (25 °C) → White (35 °C)	Thermochromic fabric	^30^
DOF/BPA/1-tetradecanol/1-hexadecanol	MF microcapsules; PDMS printing paste; cotton fabric	Red (30 °C) → Colorless (50 °C)	Temperature-visualization sensors, unidirectional moisture-wicking coatings, thermoregulatory fabric	^63^
Green fluorane/BPA/ diphenyl carbonate	PMMA microcapsules	Green (<64.4 °C) → Light yellow (>64.4 °C)	Smart fabrics, sensors, encrypted codes	^59^
JYGreen-1/2-naphthol/methyl dodecanoate	Methylcellulose microcapsules	Black (−5 °C) → White (0 °C)	Cold chains, fabrics	^65^
Magenta fluorane/BPA/ diphenyl carbonate	PMMA microcapsules	Magenta (<64.4 °C) → Light yellow (>64.4 °C)	Smart fabrics, sensors, encrypted codes	^59^
OBD-2/2-naphthol/methyl dodecanoate	Methylcellulose microcapsules	Blue (−5 °C) → White (0 °C)	Cold chains, fabrics	^65^
OBD-2/BPA/*n-*dodecanol/ *n*-decanol	Melamine (first shell layer)/phytic acid/chitosan (second shell layer) microcapsules; polyurea composite coating	Black (−10 °C) → Gray-white (25 °C)	Maintaining operation of outdoor industrials and infrastructure	^69^
OBD-2/BPA/1-tetradecanol	MF microcapsules with silicon carbide; PDMS printing paste; cotton fabric	Black (15 °C) → White (45 °C)	Thermal-regulating fabric with solar absorption	^62^
OBD-2/BPA/1-tetradecanol	Polyethylene glycol (PEG)/methyl methyacrylate/ethylene glycol dimethacrylate/2,2′-azobis(2-methylpropionamide) dihydrochloride microcapsules; boron nitride; PDMS	Black (20.6 °C) → White (31.3 °C)	Wearable textiles and automotive applications	^70^
Orange fluoran-based dye/BPA/*n-*hexadecanol	Silica microcapsules; acrylic fabric	Orange (15 °C) → White (45 °C)	Smart textile, thermochromic coating	^64^
Phenolphthalein/octadecylamine/ *n-*decanol	Ca^2+^ cross-linked alginate beads	Purple (0 °C) → White (5 °C)	Integrated temperature sensor, energy storage in cold chain logistics	^72^
SO/BPA/binary fatty alcohol eutectic mixture	Delignified eucalyptus fiber skeleton aerogel	Dark green (25 °C) → White (30 °C)	Home automation, passive infrastructural energy-saving, luminescent response switches	^29^
SO/BPA/binary fatty alcohol eutectic mixture	Delignified eucalyptus fiber skeleton coated with PVA/PG	Light green (<38 °C) → Dark green (>38 °C)	Building energy saving, intelligent windows, decoration	^28^
SO/phenolphthalein/ *n-*tetradecanol	PMMA nanocapsules	Green (<33 °C) → White (>33 °C)	Thermal energy storage material	^49^
TFG/2-naphthol/methyl dodecanoate	Methylcellulose microcapsules	Green (−5 °C) → White (0 °C)	Cold chains, fabrics	^65^

### 4.1 Thermochromic Microcapsules

Geng et al. (2018) developed thermochromic microcapsules using methylated melamine-formaldehyde as the shell and a DDS core consisting of CVL, BPA, and 1-tetradecanol.^38^ Thermal analysis showed that the incorporation of DDS components reduced the crystallinity of 1-tetradecanol, leading to lower phase-change enthalpy and crystallization temperature. Increasing emulsifier content reduced particle size but did not significantly affect shell thickness, which was attributed to increased viscosity and possible self-polymerization during shell formation. Smaller particle sizes resulted in reduced color intensity, likely due to restricted crystallization of the solvent within confined spaces. Increasing the core-to-shell ratio enhanced color intensity by improving solvent crystallization but reduced thermal stability due to thinner shells. At higher shear rates, the microcapsules exhibited smaller particle sizes and a narrower size distribution but also showed irregular morphology and rough surfaces. The microcapsules demonstrated good durability over 100 thermal cycles, with only minor structural deformation observed. Importantly, the thermochromic microcapsules were successfully applied in firefighter protective clothing, where they provided both thermal insulation and a visual indication of high temperatures. However, the study highlights a key limitation of microencapsulation, where confinement effects and structural trade-offs between particle size, morphology, and shell integrity can compromise color intensity and thermochromic responsiveness.

Building on the limitations of conventional microencapsulation systems, particularly the confinement effects and instability observed by Geng et al.[Bibr ref38] (2018), Pu and Fang (2022) developed thermochromic microcapsules using methylcellulose as a shell material to improve encapsulation stability.^48^ The DDS core, consisting of CVL, dodecyl gallate (DG), and methyl dodecanoate, exhibited a color transition from blue at 0 °C to colorless at 10 °C. The use of methylcellulose improved dispersion and prevented phase separation, which was previously observed in nonencapsulated systems, indicating enhanced stability of the thermochromic compound. In addition, the microcapsules showed more uniform coloration and eliminated the formation of crystalline residues, suggesting improved encapsulation efficiency. However, the study revealed a new limitation related to composition control. Increasing the developer content hindered discoloration at higher temperatures due to insufficient solvent to disrupt dye–developer interactions, while low dye and developer concentrations resulted in weak or absent coloration at low temperatures. This indicates that, despite improvements in encapsulation stability, thermochromic performance remains highly sensitive to the balance of DDS components. Furthermore, the overall color difference decreased after encapsulation, highlighting a trade-off between stability and visual intensity.

Özkayalar et al. (2020) used phenolphthalein as an alternative developer to achieve thermochromism in DDS compounds.^49^ Coupled with 2′-dibenzylamino-6′-diethylaminofluoran leuco dye and n-tetradecanol cosolvent, the developed compound changes from green to colorless when heated. They also encapsulated the compound with polymethylmethacrylate (PMMA). Spherical nanocapsules with a lower average diameter of approximately 200 nm were fabricated. The addition of 2,4-dihydroxybenzophenone enhanced the UV resistance of the samples, on top of it having developer-like properties. Color changes from green at room temperature to white above 33 °C were observed. The melting temperature of the cosolvent was found to decrease when added to the thermochromic system and decreased further in nanocapsules. The thermochromic compound with and without the capsule shell was found to have melting enthalpies of over 200 J g^–1^ and 180 J g^–1^, making them suitable for energy storage.

Further advancing microencapsulation strategies, Jiang et al. (2023) developed thermochromic microcapsules using a silica shell to enhance thermal stability and heat transfer properties.^50^ Compared to polymer-based shells, the inorganic silica shell provided improved thermal conductivity, enabling faster heat transfer and more responsive thermochromic behavior. The DDS core, composed of CVL, BPA, and *n-*hexadecanol, was successfully encapsulated, and the resulting microcapsules exhibited good durability and structural stability over repeated thermal cycles. In addition to improved stability, the study showed that increasing BPA content enhanced color brightness and intensity, indicating stronger dye–developer interactions. However, similar to previous studies, the performance remained highly dependent on DDS composition. Insufficient solvent content limited the dissociation of dye–developer interactions, resulting in incomplete discoloration. Furthermore, despite the improved thermal conductivity of the silica shell, encapsulation still reduced melting enthalpy and broadened the phase transition range due to confinement effects within the microcapsules. These findings suggest that while the use of inorganic shells can improve thermal response and stability, fundamental limitations associated with confinement and phase behavior of the DDS core persist. Therefore, enhancing shell properties alone is insufficient to fully overcome the intrinsic trade-offs in microencapsulated thermochromic systems.

Yang et al. (2023) further investigated the influence of structural parameters by varying the core-to-shell ratio in melamine–formaldehyde microcapsules containing CVL, BPA, and *n-*hexadecanol.^42^ Increasing the core content enhanced color intensity due to improved crystallization of the solvent phase. However, this also reduced shell thickness, resulting in poorer leakage resistance and reduced thermal stability. This study highlights that core-to-shell ratio is a critical design parameter governing the trade-off between thermochromic performance and structural integrity, reinforcing the need for balanced microcapsule design.

Wang et al. (2023) examined the effect of encapsulation on the thermal properties of DDS systems using melamine–urea–formaldehyde shells with a CVL/BPA/*n-*decanoic acid core.^51^ They reported a reduction in both phase-transition temperature and enthalpy after encapsulation, attributed to restricted molecular mobility within the confined structure. Despite this, encapsulation improved thermal stability and reduced volatilization of core components. These findings confirm that while encapsulation enhances durability, it inherently alters phase-change behavior, further emphasizing the influence of confinement on thermochromic performance.

Moving beyond structural optimization, Wu et al. (2023) introduced compositional tuning through the use of a binary eutectic mixture of *n*-tetradecane and *n*-dodecanol within melamine–formaldehyde microcapsules.^25^ The eutectic composition exhibited the lowest melting enthalpy and a narrower phase transition range, demonstrating improved control over switching temperature. However, increasing core content reduced encapsulation efficiency and led to leakage, while variations in emulsifier composition affected particle size and color uniformity. These results indicate that although compositional design provides additional flexibility in tuning thermochromic behavior, it introduces further complexity in balancing stability, encapsulation efficiency, and optical performance.

Liu et al. (2025) fabricated thermochromic CVL/BPA/*n-*tetradecanol microcapsules with calcium alginate as the shell material.^52^ The microcapsules were found to be spherical, with a particle size range of 2–3 mm. The shell material effectively sealed the DDS compound within, preventing leakage during the phase transition. It was found that a moderate water/oil ratio (1:7), high sodium alginate content (2 wt %), and large needle diameter (2 mm) produced the most optimal phase-change enthalpy and thermal conductivity. Thermal cycling stability was maintained after 200 cycles. The ability for photothermal conversion makes the prepared microcapsules suitable for solar-thermal applications.

Liu et al. (2025) prepared microcapsules containing a CVL/bisphenol AF (BPAF)/*n*-butyl stearate core and silane-terminated polyurethane shell.^53^ The interference of the isocyanate groups on the color-changing properties was negated by silane termination of the shell material. The emulsifier content and core/shell ratio were found to affect the microcapsule morphology, particle size distribution, and phase change properties. The phase change enthalpy was found to be 122.2 J g^–1^, and the thermal stability, thermal cycling stability, and energy storage efficiency were found to be excellent. Hence, the thermochromic microcapsules are suitable for use in wearable electronic devices, smart material coating, and temperature-sensitive medical devices.

Liu et al. (2025) developed thermochromic melamine-formaldehyde (MF) microcapsules with CVL dye, BPAF developer, and several alkyl benzoates as the cosolvent.^54^ They found that the thermal stability of the compound significantly improved along the increasing alkyl chain length in the cosolvent. The microcapsules possessed spherical morphology and good energy storage efficiency (greater than 83%). Besides that, the samples also exhibited memory thermochromic response, which is the phenomenon of color reversion only after it is cooled to below the cosolvent melting temperature. This was attributed to the presence of a benzene ring in the molecular structure of cosolvent, as well as the spatial confinement within the microcapsules, making it difficult for crystallization to occur at its melting temperature range.

Liang et al. (2026) incorporated thermochromic microcapsules into silicone electronic potting materials to provide early warning signs of overheating for lithium batteries and integrated electronic devices.^55^ The thermochromic microcapsules were composed of a CVL/BPA/*n-*octadecanol core DDS compound in PMMA shells. Similar to previous studies, the capsule shells provided weathering resistance and improved thermal stability to the core compound. The composite was able to change color reversibly while providing temperature reduction for the tested lithium battery pack, giving visual feedback on top of thermal management.

Liang et al. (2026) incorporated thermochromic microcapsules into silicone electronic potting materials to provide early warning signs of overheating for lithium batteries and integrated electronic devices.^55^ The thermochromic microcapsules were composed of a CVL/BPA/*n-*octadecanol core DDS compound in PMMA shells. Similar to previous studies, the capsule shells provided weathering resistance and improved thermal stability to the core compound. The composite was able to change color reversibly while providing temperature reduction for the tested lithium battery pack, giving visual feedback on top of thermal management.

Wang et al. (2026) developed visually enhanced thermochromic microcapsules with photothermal properties by incorporating CVL dye, BPA developer, *n*-nonanoic acid cosolvent, and organic–inorganic functional regulating components, which are gallic acid and nano-TiO_2_.^56^ Upon encapsulating the core thermochromic compound with a melamine-urea-formaldehyde (MUF) shell, the microcapsules showed rough and undulating surfaces rather than smooth spherical structures, indicating that the functional components altered the deposition behavior of shell materials. Moreover, the introduction of functional components was found to greatly enhance the visibility of the thermochromic response at the cost of some of the latent heat storage capacity, while the photothermal cycling stability, up to 10 cycles, was not significantly affected. From the results, the authors concluded that the modifications done to the thermochromic system should be viewed as an application-oriented case due to the trade-off between visual and thermal storage performance.

Collectively, these studies show a shift from improving encapsulation techniques to optimizing the structural and compositional parameters of microcapsules. While parameters such as the core-to-shell ratio, shell material, and eutectic composition provide additional control over thermochromic behavior, they also introduce competing effects between stability, crystallinity, and color performance. This highlights that microcapsule design is inherently a multiparameter optimization problem, where improvements in one aspect often lead to compromises in another.

The interdependence between encapsulation parameters and thermochromic performance is summarized in Figure 3, which illustrates the trade-offs between color intensity, response speed, and stability.

**Figure 3:**
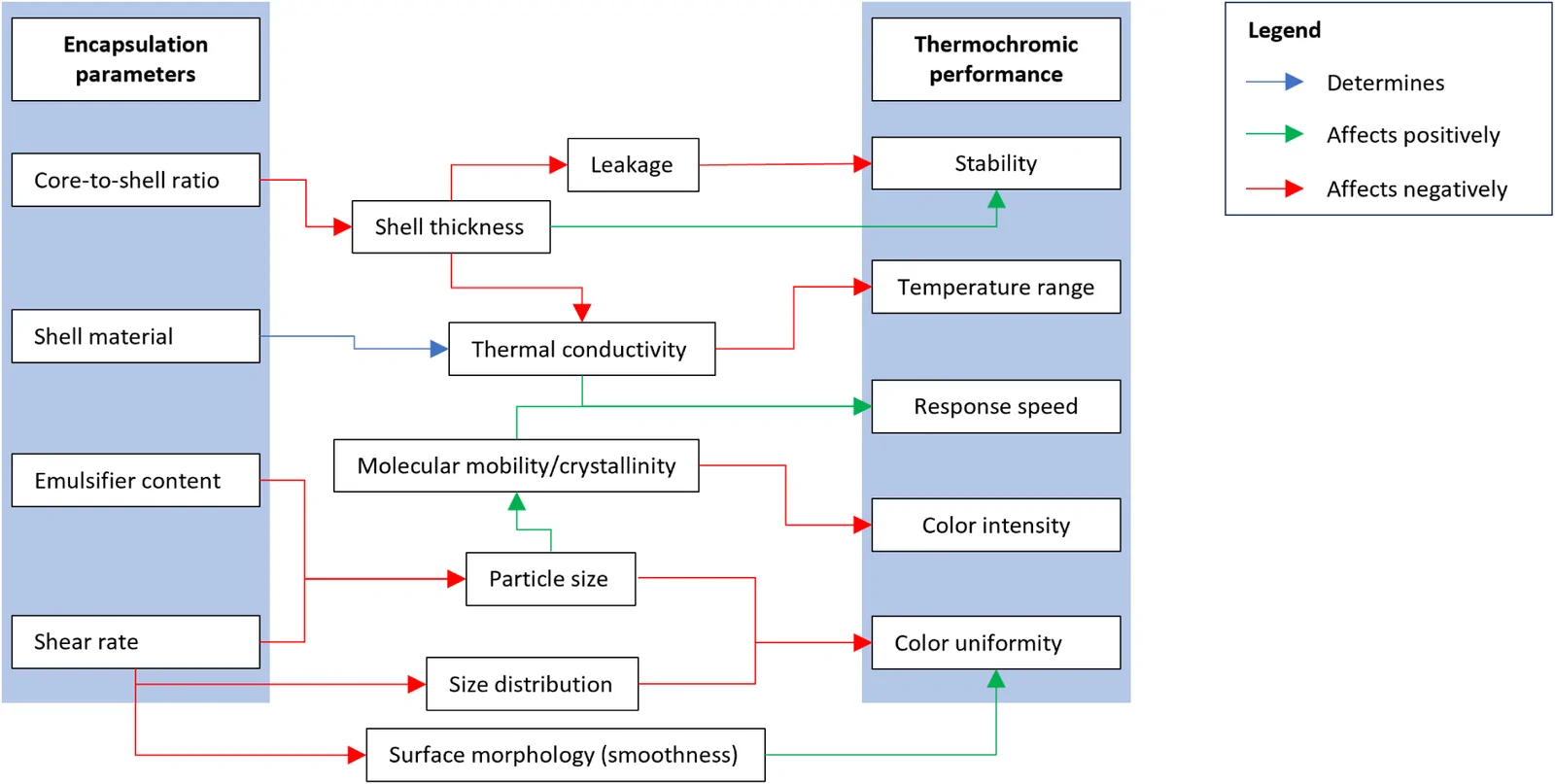
Balance and trade-offs in thermochromic microcapsules.

### 4.2 Thermochromic Wood Composites

Tao et al. (2023) developed thermochromic eucalyptus wood aerogel composites using the DDS system with a binary eutectic mixture of fatty alcohols.^29^ The samples exhibit a red/green to milky white color change upon heating, accompanied by a change in material flexibility from rigid to flexible. The incorporation of dye and developer was found to reduce the phase transition temperature, which decreased due to the disruption of solvent crystallization within the porous structure. This indicates that the porous matrix facilitates rapid heat transfer and also alters the thermodynamic behavior of the DDS system.

Similarly, Kang et al. (2023) produced flexible thermochromic eucalyptus wood films with 3,3′-bis­(1-n-octyl-2-methylindol-3-yl)­phthalide (BP) or 2′-(dibenzylamino)–6′-(diethylamino)­spiro­[isobenzofuran-1­(3H),9′-[9H]­xanthene]–3-one (SO), BPA, and a binary eutectic mixture of two of three fatty acids: *n-*tetradecanoic acid, *n-*hexadecanoic acid, and *n-*octadecanoic acid.^28^ The composites showed a distinct color transition from light red/green to dark red/green when heated. The incorporation of thermochromic wood with poly­(vinyl alcohol) (PVA)/propylene glycol (PG) resulted in red and green wood composites that showed good thermal cycling stability up to 50 cycles. However, the incorporation of DDS compounds reduced thermal stability, suggesting that interactions between the thermochromic system and the wood matrix may compromise material durability.

Zhu et al. (2024) further demonstrated thermochromic Hinoki wood composites by embedding encapsulated DDS systems within a PVA coating on delignified wood.^57^ The materials exhibited a rapid color response within seconds, attributed to efficient heat transfer through the thin coating layer. In addition, the thermochromic layer provided thermal storage capability due to latent heat absorption during the phase transition. However, an uneven distribution of microcapsules within the wood structure limited the uniformity of the thermochromic response, highlighting challenges in achieving consistent performance in porous substrates. On the basis of the results and observations, the authors proposed low-temperature thermal storage applications for the developed thermochromic wood.

Song et al. (2025) tackled the flame susceptibility of thermochromic wood composites by embedding nano-CaCO_3_.^58^ The developed wood composites exhibited reversible thermochromic properties as well as good thermal energy storage and thermal stability. When burned, the wood composites with nano-CaCO_3_ showed a dense layer of carbon on the surface, preventing the penetration of oxygen and heat transfer through the pores that would be present in wood without the additive, thus imbuing the composite with flame-retardant properties. With the improved shelf life, the wood composite was expected to store thermal energy well as information storage, decoration, and furniture.

### 4.3 Thermochromic Fabrics

Wang et al. (2022) developed thermochromic fabrics by screen printing microcapsules containing fluorane dyes, BPA, and diphenyl carbonate.^59^ The magenta fluorane dye underwent reversible transition from magenta to light pink/light yellow between 64.4 and 66.5 °C, while the green dye changed from green to light yellow within the same range. In the absence of a color developer, no thermochromic behavior was observed, confirming the critical role of developer–dye interaction. The synthesized fluorane dyes exhibited higher temperature sensitivity compared to conventional dyes. Particle size, controlled by emulsification rate, had little effect on color depth or phase transition temperature; however, smaller particles increased color hysteresis due to restricted crystallization. Thicker shells reduced color intensity and broadened the transition range, attributed to lower thermal conductivity. The fabrics demonstrated multistage color transitions (36–39 °C), indicating potential for smart textiles, temperature sensing, and encryption. However, confinement effects and shell thickness remain key factors limiting color clarity and response precision.

Xue and Zhao (2023) developed electrothermal color-changing fabrics by coating cotton with graphene on the backside and thermochromic paste on the front.^40^ The DDS system (CVL/BPA/1-tetradecanol-1-octadecanol) with carbon nanotubes (CNTs) was encapsulated in melamine–formaldehyde shells. The incorporation of CNTs increased particle size and reduced melting enthalpy, while also improving thermal conductivity and narrowing the color transition range. Supercooling was observed after encapsulation due to reduced nucleation sites, but could be mitigated by CNT addition or by adjusting phase change components. The system exhibited a reversible blue-to-white transition between 20–37 °C, both thermally and under electrical stimulation (6 V). The fabrics showed excellent durability over 500 cycles and effective thermal regulation due to latent heat absorption. However, increasing CNT content introduced trade-offs, including reduced phase change efficiency and altered thermal behavior.

Yang et al. (2024) incorporated CVL/BPA/*n-*tetradecanol thermochromic compound with an MUF capsule shell into polyethylene terephthalate (PET) fabrics, along with humidity-sensitive discoloration materials (HDMs).^60^ The microcapsules changed from blue to white between temperatures 31 and 45 °C. With the HDMs alone, the fabric showed color variations from yellow to orange when the humidity was reduced from 100% to 0%. By combining thermochromic microcapsules and HDMs, the fabric showed an even wider range of color variation with temperature and humidity. From 30 to 80 °C, the color changed gradually from green to yellow, to orange to red. Whereas, from 0% to 100% humidity, the fabric was seen to change from purple to red and then to green. The color changes for 30 cycles were found to be consistent. With the dual-responsive function, the fabrics were proposed to be used in fashion, environmental monitoring, healthcare, and information encryption.

Yu et al. (2024) developed double-stage thermochromic fabrics using two DDS systems (DDF/BPA/*n-*tetradecanol and CVL/BPA/*n-*hexadecanol) encapsulated in melamine–formaldehyde.^30^ The optimized emulsifier content (0.9 wt %) ensured stable microcapsule formation. The fabrics exhibited distinct color transitions at multiple temperatures, showing pink at 20 °C, blue at 30 °C, and white at higher temperatures. The phase-change temperatures were lower than pure solvents due to disruption of crystallization by the dye and developer components. The fabrics demonstrated good durability and solvent resistance, with stable performance over repeated cycles. However, overlapping response ranges between the two systems may limit precise control of multistage color transitions.

Zhou et al. (2024) developed thermochromic microcapsules using CVL, BPA, and dodecyl decanoate encapsulated in melamine–formaldehyde.^46^ The system exhibited a reversible color change from blue to yellow within the 20–35 °C range. Encapsulation slightly reduced melting enthalpy but improved thermal stability by protecting the core material. When applied to cotton fabric, the microcapsules showed rapid and reversible thermochromic response, maintaining performance over 90 cycles and showing minimal degradation after 120 cycles. These results highlight the effectiveness of encapsulation in improving durability, although confinement effects continue to influence phase-change behavior.

Tözüm (2025) fabricated PMMA microcapsules containing a thermochromic compound with CVL dye, phenolphthalein developer, and 1-tetradecanol cosolvent.^61^ The microcapsules were spherical in shape and had an average particle size of 19.17 μm. The observed color change was from blue to colorless at temperature range of 20–50 °C. Energy storage properties were found in the microcapsules (219.5 J g^–1^), owing to the presence of 1-tetradecanol. The microcapsules were then attached onto fabric, which retained the thermochromic properties. The pores on the fabric were filled by the microcapsules, which significantly decreased the permeability. Moreover, the bending resistance was increased and the tear strength was decreased. The author suggested that smart textiles, stimulant and protective clothing, aerospace engineering, and wearable electronic systems would be potential applications.

Xue et al. (2025) developed black-white thermochromic cotton fabrics using 2-anilino-6-(dibutylamino)-3-methylfluoran (OBD-2)/BPA/1-tetradecanol core encapsulated in MF.^62^ The addition of silicon carbide (SiC) enhanced thermal conductivity and photothermal properties while mitigating supercooling phenomena. The microcapsules with and without SiC showed similar melting enthalpy. Thermochromic fabric was produced by through screen printing polydimethoxysilane (PDMS) paste embedded with the microcapsules. The fabric changed reversibly from black to white at 20 °C and had photothermal conversion capability. Thus, the author proposed the use of the developed fabric as thermal regulation fabrics, photothermal storage, temperature displaym and energy-efficiency applications in buildings.

In their other study, Xue et al. (2026) prepared thermochromic MF microcapsules with two distinct colors, blue with CVL dye and red with 3′,6′-dimethoxyfluoran dye (DOF), with a BPA developer and a binary mixture of *n-*tetradecanol/*n-*hexadecanol as the cosolvent.^63^ The different dyes and solvent content did not result in significant effects on the spherical microcapsule shape, core–shell structure, and particle size distribution. The blue microcapsules discolored at 35–40 °C, while the red microcapsules turned white at 40–45 °C. In terms of thermal properties, both samples had decent phase-change properties and good thermal stability. The color changes were consistent for 100 thermal cycles. When the microcapsules were fixated onto cotton fabrics through screen printing, the fabric was able to inherit some of the melting enthalpy of the core compounds, indicating suitability for use in thermo-regulating clothing.

Kim et al. (2026) developed an orange thermochromic acrylic fabric using a synthesized fluoran-based dye, a BPA developer and *n*-hexadecanol cosolvent encapsulated in an SiO_2_ shell.^64^ Inorganic SiO_2_ shells have superior thermal conductivity and mechanical strength compared to organic shell materials, and were found to enhance the thermal stability and thermal cycling stability of the core compound. The absorption values were found to be consistent when tested for 10 heating–cooling cycles. Next, the microcapsules were attached to acrylic fabric, and the color changed reversibly from orange at approximately 25 °C to white at approximately 45 °C. The proposed application for the fabricated thermochromic fabric is as smart textiles and thermochromic coatings.

Feng et al. (2026) used 2-naphthol as the developer, methyl dodecanoate as the cosolvent, and several dyes, namely CVL, CVL/DDF, DDF, 3,3-bis­(2-ethoxy-4-N,N-diethylaminophenyl)-7 (4)-azaphthalide (JYGreen-1), OBD-2, and 6′-(diethylamino)-2′-(phenylamino)-3H-spiro­[2-benzofuran-1,9′-xanthen]-3-one (TFG).^65^ The compounds were encapsulated with methylcellulose before being incorporated into alginate/methylcellulose fibers. The different compounds were able to show color changes within 1 °C of the subsequent one, making them good for indicating specific temperatures from 0 °C to −5 °C. The fibers exhibited reversible color change within 60 s, and the thermal cycling stability was good for more than 200 cycles. From the observed results, the authors recommend using the compounds for smart fabrics in cold-chain delivery or temperature indication in cold weather.

Yu et al. (2026) fabricated positive and negative thermochromic compounds using developers DG and BPA, respectively, along with the synthesized fluoran dye 2′-bromo-6′-diethylaminofluoran (BDF) and 1-tetradecanol as the solvent.^19^ The compound with the DG developer underwent colorless-to-red transition at 47 °C, while the compound with BPA changed from red to colorless at 35 °C. They discovered that the positive thermochromic compound (with DG) was suitable for vacuum impregnation into linen fabric, whereas the negative thermochromic compound (with BPA) need was encapsulated and printed onto cotton fabric. Furthermore, encapsulation reduces phase separation and also allows for more diverse applications. Both types of fabrics were tested to have reversible thermochromic changes and decent cycling reversibility for 50 thermal cycles, making the materials good as wearable thermochromic textiles with tunable phase transition temperature and bidirectional color changes.

### 4.4 Other Forms of Thermochromic Polymers

Zhang et al. (2022) developed reversible thermochromic silicone rubber materials that are reusable up to 30 temperature cycles.^66^ The core compound, consisting of CVL, BPA, and phase-change wax, was encased in silica shell and the resulting microcapsules were added into silicone rubber. While the addition of CVL and BPA did not significantly affect the thermal stability of phase-change wax, the melting and crystallization temperatures were lowered due to disruptions in the crystalline structure. The particle size of the microcapsules was in the range of 100–300 nm. Encapsulation reduced the melting enthalpy and improved the thermal stability of the thermochromic compound while maintaining clear color changes from dark blue at 25 °C to light blue or white at 40 °C that are observable through the white silica shell. A higher developer content was reported to give the microcapsules a deeper color, thus enhancing observability of color change, as well as the sensitivity and effectiveness of the thermochromic response. The incorporation of the microcapsules into silicone rubber was found to increase the tensile strength and fracture toughness, which was attributed to more effective stress distribution from improved matrix–filler and matrix–matrix interactions. These observations were also accompanied by an increase in elongation at break, brought about by the deflecting of crack propagation in the silicone rubber matrix by the microcapsules. Morphological studies showed that the microcapsules were uniformly dispersed in the middle of two silicone rubber layers, thus preventing migration of the microcapsules while maintaining the inherent transparency and hydrophobicity of the silicone rubber matrix. The core compound possessed rapid energy storage and slow energy release, which could be useful in thermal insulation applications, while the silicone rubber composite was proposed to be used as temperature-sensitive display and wearable electronic devices.

Shayan et al. (2024) fabricated nanofiber mats using CVL/BPA/1-dodecanol as the core and polylactic acid (PLA)/modified cellulose nanocrystals as the shell.^67^ They reported that the mechanical properties were affected by the addition of core materials, attributed to the poor interaction between the core and shell materials. The glass transition temperature was found to be lower in thermochromic PLA fibers, mainly due to the presence of 1-dodecanol as a plasticizer. Similarly, the thermal degradation onset also shifted to a lower temperature, which was recorded to be approximately 130 °C. The nanofiber mats were observed to change from dark blue at around 0.5 °C to white at 20 °C. As the temperature rises, the CIELAB coordinates correspond to the color changes; L* and b* values increased, while a value mostly remained the same, indicating that the sample became lighter and less bluish. The developed thermochromic compound was proposed to be used as temperature sensors in advanced materials science.

Xu et al. (2022) produced thermochromic filter paper by vacuum impregnation of filter paper with thermochromic compound CVL, BPA, and ethyl stearate or butyl stearate.^9^ They reported that ethyl stearate and butyl stearate acted as the component that stores thermal energy during phase change. Developer BPA has been found to affect the crystallization behavior of ethyl stearate and butyl stearate, reflected by a significant decrease in latent heat and phase-change temperature. The fabricated thermochromic filter papers were reusable after thermal cycling, and there was no leakage of the thermochromic compounds from the filter paper. The samples have good thermal stability; TGA results showed a two-step degradation that occurs at 160–245 °C and 325–380 °C, which can be attributed to the degradation of ethyl/butyl stearate and filter paper, respectively. For the application, QR codes created from the thermochromic compounds have shown good viability in use as temperature-controlled information storage.

Hu et al. (2025) fabricated thermochromic water- and oil-proof cardboard coated with sodium alginate, hydroxypropyl methylcellulose, polyvinyl butyral, and ethyl cellulose.^68^ The thermochromic compound, consisting of CVL, BPA, and *n-*octadecanol was applied to the backside of the cardboard after the coating process was done. The compound was found to change from dark blue at 30 °C to colorless at 55 °C in less than 1 min, which was claimed to be better in terms of temperature range and thermochromic performance compared to six other thermochromic materials from other works. The targeted application is fast-food packaging with some added functionalities; an aesthetic feature that also allows the consumer to gauge the temperature of the contained food before eating. The authors suggested that future research explore other nontoxic and less costly alternatives for the thermochromic compound and coating, respectively, as well as assess the long-term performance of the developed temperature-sensing water-/oil-proof cardboard.

Lin et al. (2024) prepared a dual-functional polyurea coating with flame-retardant and solar deicing properties.^69^ The core compound used was OBD-2/BPA/*n-*dodecanol/*n*-decanol and was encapsulated with two layers of shell, the first one being melamine and second layer being chitosan/phytic acid. The chitosan/phytic acid shell serves as the main flame-retardant agent, with chitosan forming the char and phytic acid promoting the formation of carbon residue. Unlike other studies, the char layer formed is sparse, limiting flame retardancy from this mechanism. In addition to that, the melamine layer also contributes to reduce the concentration of combustible gases by releasing ammonia and nitrogen from burning. As of the deicing performance, the incorporation of the microcapsules has imbued polyurea with photothermal properties. This property can absorb light sources and convert it into heat, thus melting the ice on the surface of the thermochromic polyurea coating. The coating changes between black at −10 °C and white at 45 °C. From all the above observations, the authors suggested applying the coating to outdoor industrial and infrastructure for locations with extremely dynamic weather conditions.

Fei et al. (2026) developed a similar thermal regulating compound using an OBD-2/BPA/1- tetradecanol core in microcapsules blended with boron nitride (BN) and PDMS to form a coating.^70^ BN was selected as the filler for this coating due to its exceptional solar-scattering capability, chemical stability, and decent compatibility with polymer matrices. The resultant coating changes reversibly between black (∼20 °C) and white (∼31 °C), absorbing solar radiation and converting it to heat energy during colder weather, and vice versa. The coating also has good thermal insulation, allowing for excellent heat retention. As such, the proposed applications for the developed coating are wearable textiles and the automotive industry.

Zhai et al. (2024) fabricated a thermochromic membrane with a PVA/water-based polyurethane (WPU) matrix and CVL/BPA/*n-*tetradecanol thermochromic compound.^71^ The thermochromic compound was fixed onto *n-*octadecane nanocapsules, and then the product was added to the PVA/WPU matrix. The compound changed from blue at 15 °C to white at 40 °C. With an increase in *n-*tetradecanol solvent content, the discoloration time decreased, but the recovery time increased. The optimal CVL/BPA/*n-*tetradecanol mass ratio in the compound was found to be 1:3:50, determined by a balance between short discoloration and recovery times. The nanocapsules showed a uniform spherical shape, and the thermochromic layer added to the particle size. In the thermochromic membrane, although a higher nanocapsule content caused agglomeration of particles and produced a rougher membrane surface, the nanocapsules were successfully embedded into the matrix without any chemical reactions. The nanocapsules were also able to impart heat storage capacity to the PVA/WPU membranes while not having significant effects on thermal stability. However, the tensile strength of the thermochromic membrane was found to decrease due to stress concentration and initiation of cracks by the nanocapsules, needing the addition of hydroxypropyl methyl fiber to enhance cohesion and the mechanical properties of the membrane. The thermochromic membrane was then used to develop a medical dressing, attached to a normal wound dressing as the bottom layer and a transparent dustproof cloth as the top layer. The dressing layer distances the thermochromic layer from the wound while the dustproof cloth protects the thermochromic layer. The thermochromic wound dressing showed fast and reversible response to changes in skin temperature.

Tan et al. (2023) developed thermally responsive photonic crystals combining DDS systems with structural color materials.^13^ Negative thermochromic systems (CVL/BPA/*n*-tetradecanol) showed color fading upon heating, while positive systems (CVL/DG/*n*-tetradecanol) exhibited intensified coloration. The integration with photonic crystals enabled angle-dependent color effects, which enhanced visual functionality. These systems demonstrated excellent durability, withstanding up to 2000 thermal cycles and prolonged heating without significant degradation. Applications include anticounterfeiting, information encryption, and smart windows. However, the complexity of multilayer structures may limit scalability and manufacturing feasibility.

Thanaritthimanomai and Monvisade (2025) developed thermochromic phase-change beads using phenolphthalein/octadecylamine/*n-*decanol as the thermochromic compound and Ca^2+^-cross-linked alginate as the bead material.^72^ The optimized beads were found to be uniformly spherical with diameter range of 1.8–2.2 mm and possessed a rapid and reversible thermochromic response at 25 °C, which were purple to pale pink (within 10 s) and to pale-white z9within 60 s). The thermochromic mechanism between the phenolphthalein dye and octadecylamine developer was proposed to be the same as that between CVL and BPA. When *n-*decanol melts, it creates a nonpolar environment that breaks up the dye–developer interaction. This causes closing of the lactone ring in phenolphthalein, making it appear colorless. In reverse, when *n-*decanol crystallizes, the environment becomes polar, allowing dye–developer interactions to form. The lactone ring in phenolphthalein opens, and a purple coloration is observed. The melting temperature and latent heat were 4.9 °C and 200.05 J g^–1^ respectively, indicating good thermal storage ability. This is supported by the ability of the beads to maintain a stable temperature range for 28 min. The thermochromic properties remained consistent for 500 thermal cycles while the thermal properties decreased slightly after the 100th cycle. From the thermal buffering and thermochromic capabilities, the authors proposed that the beads be used in refrigerated packaging in cold-chain logistics.

### 4.5 Comparison between Preparation Methods of Thermochromic Polymers

Table 5 shows a summary of the strengths and limitations of encapsulation and impregnation into porous matrices of thermochromic DDS compounds. Encapsulation provides protection against heat and the environment,^55^ keeps the compound sealed within a tight space,^19^ and makes it easier to handle during further processing.^54^ This makes it suitable for fabricating ready-made thermochromic dyes for embedding into polymer matrices or for adsorption onto fabrics. However, the capsule shell may become hazy and make color changes less perceivable, sometimes even interfering with the functionality of the thermochromic compound.^19^ Therefore, it is crucial to fine-tune the encapsulation parameters (Figure 3) to obtain thermochromic microcapsules with the most optimal color intensity and thermal properties.^25,42^

**Table 5:** Strengths and Limitations of Preparation Methods of Thermochromic Polymers

Preparation method	Strengths	Limitations
Encapsulation	• Improves weathering resistance and thermal stability of thermochromic compound.^55^	• Interferes with thermochromic mechanism.^19^
	• Provides strong spatial confinement.^19^	• Requires finetuning of parameters to achieve good encapsulation and other resultant properties, such as color uniformity and intensity.^25,42^
	• Allows for a wide range of applications.^54^	
	• Ease of handling during further processing.^54^	
Impregnation into porous matrices (wood)	• Renewable and low cost materials.^31,57^	• Reduction in thermal stability of porous material.^28^
	• Good shape stability.^58^	• Porous material hinders the crystallization of solvent, lowering phase transition temperature and latent heat storage.^29^
	• Furniture can double as temperature indicators and decoration.^31^	• Poor distribution of thermochromic compound in porous material.^57^
		• Requires an encapsulating matrix to prevent leakage of thermochromic compound from the material.^28^

Compared with encapsulation, impregnation of DDS compounds directly into porous wood converts the material into a thermochromic bulk material. Wood is cheap, renewable, and has good shape stability, making it easily available and reliable in terms of mechanical strength.^31,57,58^ Furniture made of thermochromic wood can not only be used to store items but also functions as a temperature sensor or serve a decorative purpose from the changes in color.^31^ On the contrary, the incorporation of DDS compounds into wood may reduce its thermal stability.^28^ Similarly to encapsulation, the porous wood matrix may cause problems in the embedded thermochromic compound, affecting the crystallization behavior.^29^ The compound may also have reduced color change visibility due to poor distribution in the wood matrix.^57^ To prevent leakage of the thermochromic compound, an encapsulation layer is required to be applied onto the wood.^28^

## 5 Progress in Developing Non-Toxic Thermochromic Polymers

As seen in the previous section, the components used in typical thermochromic DDS compounds are mostly synthetic materials, limiting the application of these compounds in the fields of food packaging and healthcare devices. Regarding finding nontoxic pigments that are especially safe for food applications, plenty of studies have been done to provide substitutes to synthetic dyes with materials such as plant-based colorants.^15^ Moreover, the inherent toxicity in synthetic materials has prompted the usage of alternative developers such as alkyl gallates.^18^ For instance, BPA was found to cause endocrine disruption in humans,^75,76^ while CVL had high potential for the same harm.^77^ Phenolphthalein, while being able to function as either the dye or developer, was banned by the US Food and Drug Administration due to potential carcinogenic properties.^78^ 2-Naphthol can cause blood circulation problems and damage the liver and kidneys.^79^

Fatty acids,^80,81^ fatty alcohols,^82^ *n*-alkanes,^83^ and alkyl benzoates,^82^ which were some of the cosolvents used in previous studies (Table 4), were found to have low toxicity and are therefore suitable in low- or nontoxic DDS compounds. In addition, using ingredients from natural resources also allows renewability through farming. All of these factors allow for the development of sustainable materials, which are renewable and environment-friendly. Table 6 lists examples of thermochromic compounds that have been produced in studies moving toward low or nontoxicity in thermochromic polymers. Due to the limited number of studies done on safe and eco-friendly DDS compounds, thermochromic polymers that involve hydrogen-bonding and pH-change thermochromic mechanisms are also featured in this section to provide a comprehensive overview on the future of nontoxic thermochromic compounds.

**Table 6:** Thermochromic Compounds from Literature with Lower Toxicity Compared to Conventional DDS Compounds

Dye	Developer	Cosolvent	Polymeric material/matrix	Color change mechanism	Thermochromic properties	Proposed applications	Reference
Alizarin	-	*n-*octadecanoic acid	Poplar wood/nanosilicon dioxide/boron nitride/xanthan gum or wood wax oil	pH change	Orange (45 °C) → Yellow (70 °C)	Thermal sensor, thermal energy management	^87^
Alizarin	Tetradecyl tetradecanoate	1-hexadecanol	Poplar/pine wood/sodium methylsilicate	Proton transfer	Grayish-purple (52 °C) → Deep purple (70 °C)	Smart furniture	^43^
BCP	Octadecanoic acid	*n-*dodecanol/*n-*tetradecanol	-	Proton transfer	Dark red (<13.5 °C) → Yellow (>13.5 °C)	Thermal sensor	^21^
BCP	Octadecanoic acid	*n-*dodecanol/*n-*hexadecanol	-	Proton transfer	Dark red (<19.4 °C) → Yellow (>19.4 °C)	Thermal sensor	^21^
BCP	Octadecanoic acid	*n-*dodecanol/*n-*octadecanol	-	Proton transfer	Dark red (<21.9 °C) → Yellow (>21.9 °C)	Thermal sensor	^21^
BCP	Octadecanoic acid	*n-*tetradecanol/*n-*hexadecanol	-	Proton transfer	Dark red (<32.1 °C) → Yellow (>32.1 °C)	Thermal sensor	^21^
BCP	Octadecanoic acid	*n-*hexadecanol/*n-*octadecanol	-	Proton transfer	Dark red (<45.7 °C) → Yellow (>45.7 °C)	Thermal sensor	^21^
BCP	Tetradecanoic acid	*n-*decanoic acid	Balsa wood/silica sol; chitosan/phytic acid/MF coating	Proton transfer	Light green (16 °C) → Yellow (40 °C)	Flame-resistant construction	^94^
CPR	-	*n-*dodecanoic acid	-	pH change	Red (25 °C) → Colorless (70 °C)	Food/healthcare packaging	^17^
CPR	-	Water	Silicone microcapsule; PDMS	pH change	Pink (−80 °C) → Yellow (25 °C)	Intelligent sensors, coating and food packaging	^95^
Curcumin	-	TEA	Cellulose acetate	pH change*	Yellow (25 °C, 0 h) → Brick-red (25 °C, 24 h)	Food and pharmaceutical temperature sensor	^86^
CVL	LAP	*n-*tetradecanol	-	Proton transfer	Colorless (20 °C) → Light blue (50 °C)	Thermal sensor,heat energy storage	^12^
CVL	Tea polyphenols	*n-*tetradecanol	PDMS porous film	Proton transfer	Blue (20 °C) → White (50 °C)	Smart wearable displays	^92^
CVL; DDF; DOF; BEDA	Tea polyphenols	*n-*tetradecanol	PDMS coating; fabric	Proton transfer	Blue; red; yellow; cyan (25 °C) → White (50 °C)	Smart textiles	^91^
Cyanidin chloride	DG	*n-*hexadecanoic acid	PLA	Phase separation	Wine red (25 °C) → Violet (45 °C)	Temperature sensor	^84^
Delphinidin chloride	DG	*n-*hexadecanoic acid	PLA	Phase separation	Wine red (25 °C) → Violet (45 °C)	Temperature sensor	^85^
FLU2	Octadecyl gallate	*n-*hexadecanoic acid	PVA	Proton transfer	Colorless (<62 °C) → Red (>62 °C)	Thermal energy stoarge, textile engineering, anticounterfeiting, smart windows	^20^
Methyl red	-	*n-*decanoic acid	Poplar wood/silica sol	pH change	Light red (24 °C) → Dark red (40 °C)	Smart furniture	^88^
Methyl red	Tetradecyl tetradecanoate	-	Poplar wood/propylene wax	pH change	Light red (38 °C) → Dark red (46 °C)	Smart furniture	^31^
Methyl red	-	Tetradecyl tetradecanoate	Poplar wood/silica sol	pH change	Light red (16 °C) → Dark red (42 °C)	Smart building material	^89^
PMMA photonic crystal	-	PEG600	Polyester fabric	Refractive index change^a^	Colored (<5 °C) → Colorless (>5 °C)	Cold chain transportation	^96^
PMMA photonic crystal	-	PEG1000	Polyester fabric	Refractive index change^a^	Colored (<10 °C) → Colorless (>10 °C)	Cold chain transportation	^96^
PMMA photonic crystal	-	PEG1500	Polyester fabric	Refractive index change^a^	Colored (<20 °C) → Colorless (>20 °C)	Cold chain transportation	^96^
PMMA photonic crystal	-	PEG2000	Polyester fabric	Refractive index change^a^	Colored (<50 °C) → Colorless (>50 °C)	Cold chain transportation	^96^
Red cabbage anthocyanin	-	*n-*decanoic acid/ *n-*dodecanoic acid	Alginate hydrogel beads	pH change^a^	Colored (6 °C) → Lighter color (22 °C)	Smart food packaging	^35^
Rhodamine-B (modified)	DG	*n-*decanoic acid	PVA film	Proton transfer	Colorless (25 °C) → Pink (70 °C)	Thermal energy stoarge, textile engineering, anticounterfeiting, smart windows	^20^
Spiropyran (synthesized)	-	Aromatic solvent S1000/*n-*octadecane	Polyurethane microcapsules	Isomerization	White (30 °C) → Magenta (80 °C)	Intelligent thermal management systems	^90^
TCG	DG	Decyl decanoate	-	Proton transfer	5 and 35		^93^

a This denotes irreversible thermochromic reactions.

### 5.1 Natural Dyes and Derivatives

Natural dyes such as anthocyanins and curcumin have been widely explored as nontoxic alternatives to conventional leuco dyes. These systems rely on mechanisms such as hydrogen bonding and pH-induced structural changes rather than classical dye–developer interactions. While they offer improved safety and biodegradability, their thermochromic performance is often limited by poor thermal stability and sensitivity to environmental conditions such as pH and light.

Seeboth et al. (2013) used the natural pigment derivative cyanidin chloride as the dye, the food grade antioxidant DG as the color developer, and *n*-hexadecanol as the cosolvent to produce the first nontoxic thermochromic polymer.^84^ They proposed that the thermochromic changes observed were due to the interaction between the cyanidin chloride dye and the PLA matrix. When the sample was heated, the transition from the mobile amorphous phase to the rigid amorphous phase in the PLA matrix disrupted dye–matrix hydrogen bonding, causing thermochromic changes to occur (Figure 4). They proposed that the thermochromic pathway was either the rearrangement of PLA chains or the phase separation between the dye-rich and PLA-rich domains.

**Figure 4:**
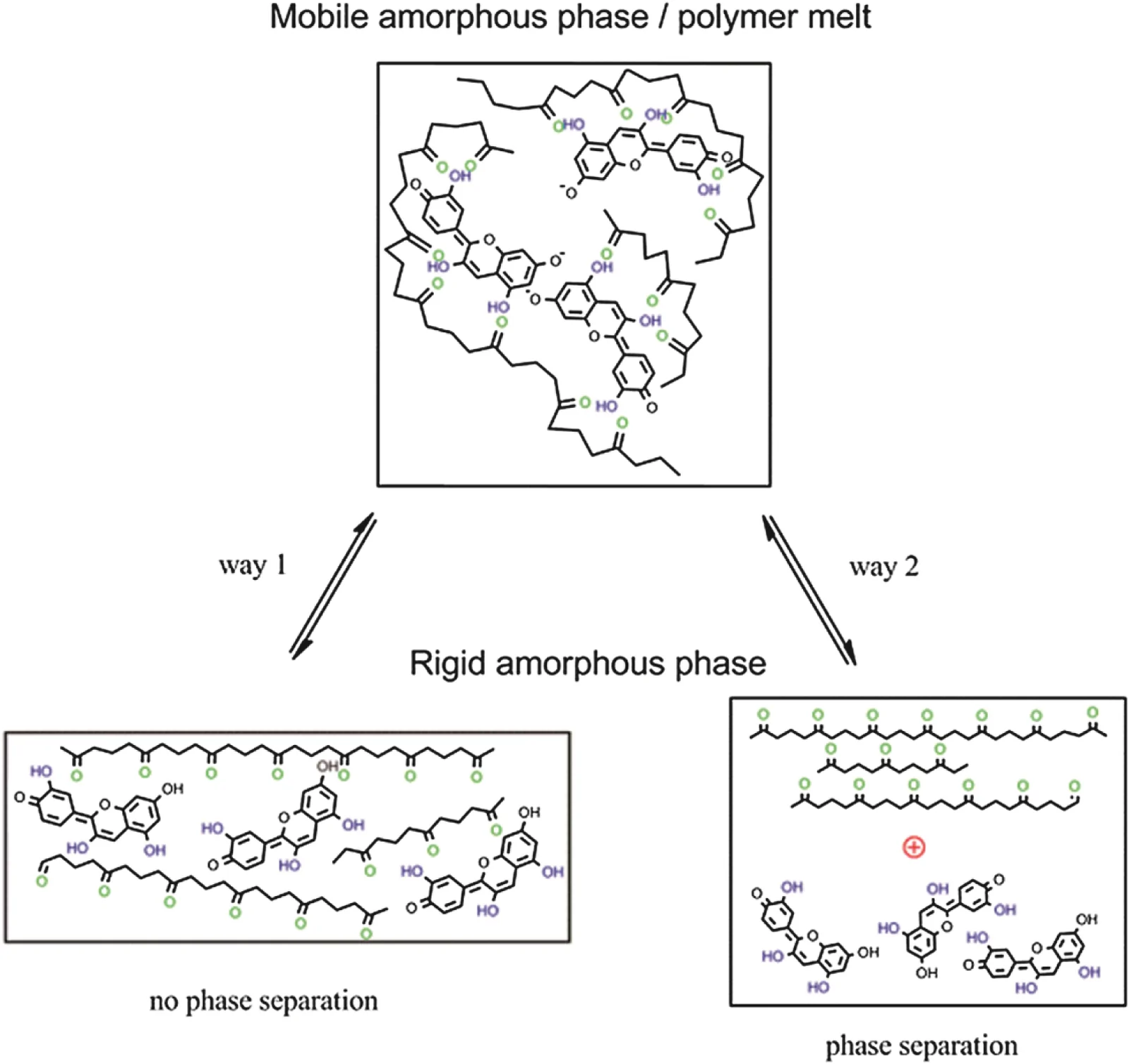
Scheme of thermochromism by interaction between PLA and cyanidin; way 1: without phase separation; way 2: phase separation.

Reproduced under terms of the CC BY license.[Bibr ref84] 2013, Seeboth et al., published by The Royal Society of Chemistry.

In a follow-up study, Lötzsch et al. (2013) investigated the influence of dye molecular structure on the ability to exhibit thermochromic behavior.^85^ Figure 5 shows the molecular structures of the anthocyanidins studied, namely pelargonidin chloride, cyanidin chloride, and delphinidin chloride. They discovered that the presence of hydroxyl groups in the B-ring of anthocyanidin was important in imparting thermochromism. Samples with cyanidin chloride and delphinidin chloride showed thermochromic properties, while that with pelargonidin did not, indicating that the presence of adjacent hydroxyl groups are crucial to impart thermochromic properties.

**Figure 5:**
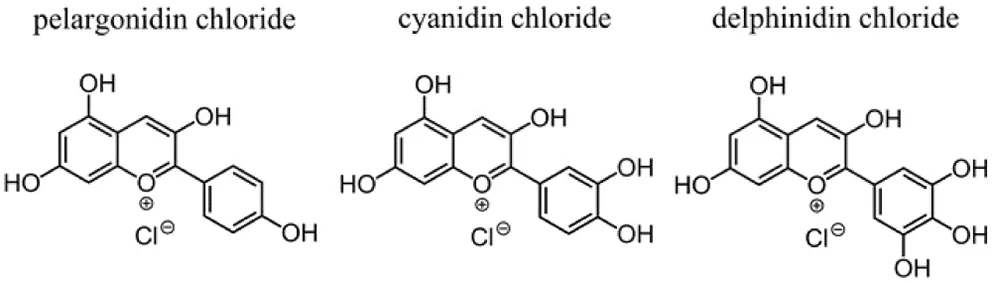
Molecular structure of the investigated anthocyanidin dyes.

Reproduced under terms of the CC BY license.[Bibr ref85] 2013, Lötzsch et al., published by Scientific Research.

Pereira et al. (2024) developed a curcumin bilayer thermochromic system. The first layer consists of curcumin extracted from rhizomes of turmeric (*Curcuma longa Linn*), cellulose acetate, and glycerol, while the second layer consists of triethanolamine (TEA) and sorbitol.^86^ The top layer changed irreversibly from yellow to brick-red when the bilayer material was exposed to temperatures above 25 °C, as shown in Figure 6. This color change was triggered when TEA in the bottom layer underwent phase change from solid to liquid, migrating into the top layer, and causing a shift toward alkaline pH. The authors noted that this color change took place over 24 h, making it suitable for monitoring the storage temperature of food or pharmaceutical products that are to be stored between 5 and 8 °C.

**Figure 6:**
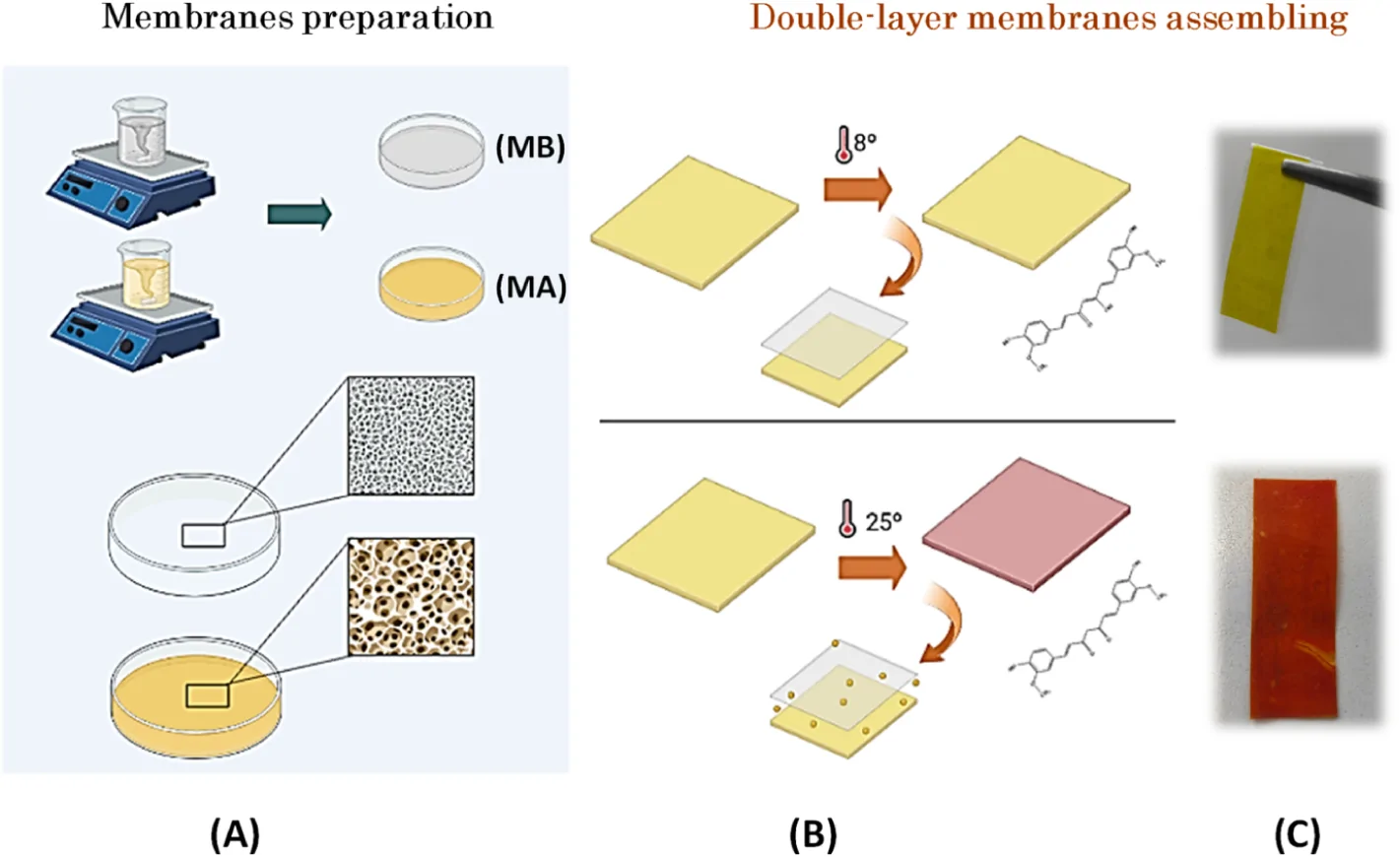
Membrane preparation: double membrane preparation (A); color development after 24 h exposed at 8 °C (upper figures (B) and (C)) and color development after 24 h exposed at 25 °C (lower figures (B) and (C)).^86^ (“Photograph courtesy of ‘Pereira et al.,’Copyright 2024”).

With a similar migration mechanism as the previous study, Raji et al. (2024) fabricated edible thermochromic beads using anthocyanin from red cabbage as the dye, a eutectic mixture of *n-*dodecanoic acid and *n-*decanoic acid as the solvent, and alginate as the main material for the core and shell.^35^ Figure 7 shows the mechanism of thermochromic color change. As the temperature was increased from 6 to 22 °C, the fatty acid mixture embedded in the core melted and migrated outward into the anthocyanin-filled shell layer. This triggered the anthocyanin to change color from the acidic pH. The diffusion of fatty acids into the outer layer was irreversible, and thus the thermochromic changes were also permanent. A few different shades of color were successfully developed for the beads before color change: green at pH 8, blue at pH 6, and purple at pH 4. After color change, the beads that were initially at pH 8 and pH 6 were found to be purple, while those at pH 4 were light pink in color. Proposed applications for the thermochromic beads are smart packaging for cold-chain fruits and vegetables.

**Figure 7:**
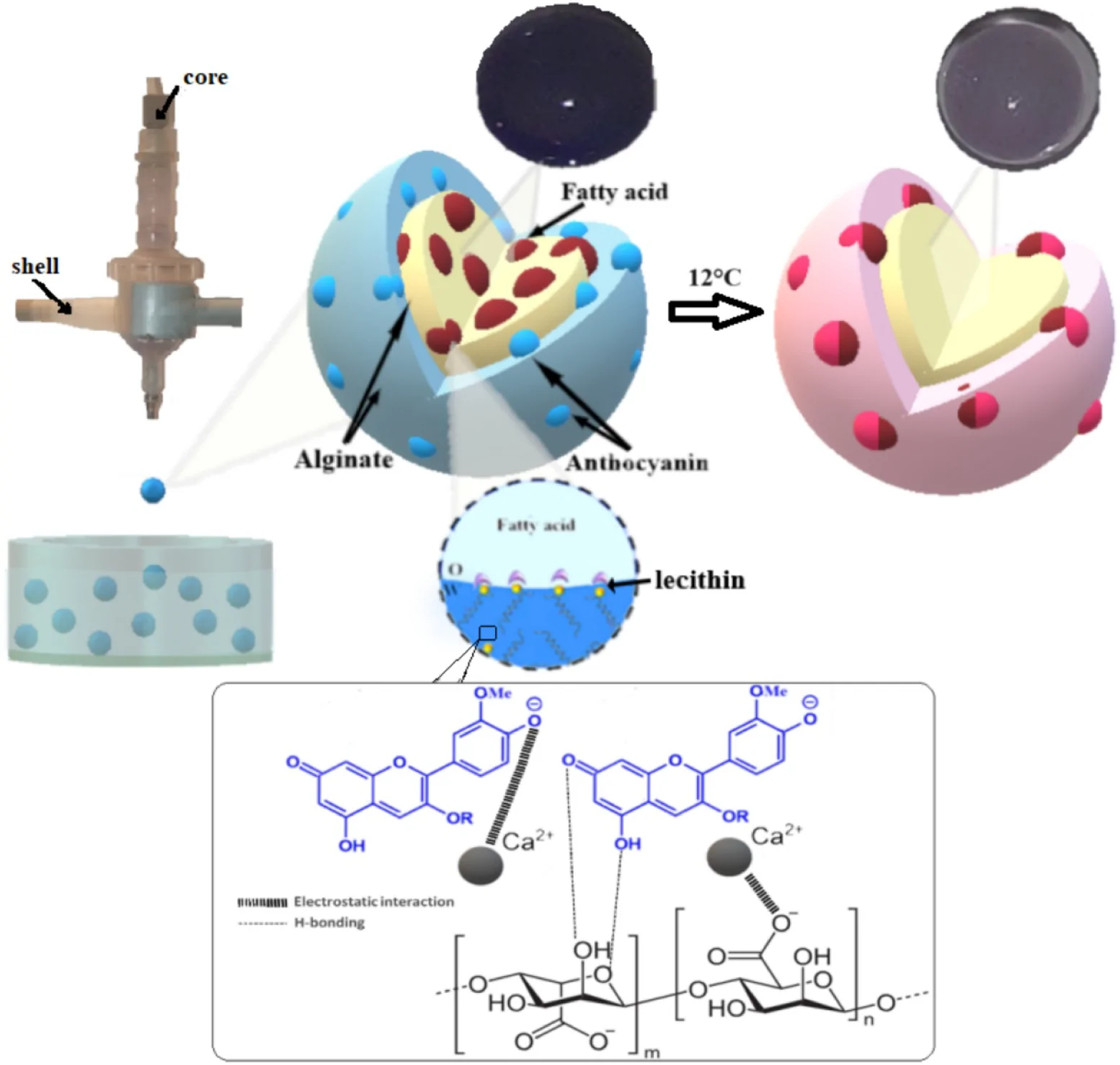
Schematic illustration for the preparation of thermochromic beads composed of fatty acid, flavonoid solutions, and alginate using a coaxial device.^35^ (“Photograph courtesy of ‘Raji et al,’Copyright 2024”).

Zou et al. (2025) fabricated thermochromic poplar wood with a binary thermochromic compound, which consists of alizarin, which is a natural pigment found in madder roots, and *n-*octadecanoic acid.^87^ Additives nanosilicon dioxide and hexagonal boron nitride were added to the wood composite, and xanthan gum and wood wax oil films were used as e encapsulation agents. Figure 8A, Figure 8E and Figure 8F show the thermochromic mechanism of plain thermochromic wood (TPCM), wood composite with nanosilicon dioxide (TPCM-Si), and wood composite with both nanosilicon dioxide and boron nitride additives (TPCM-Si-BN), respectively. Generally, an orange color is seen when alizarin contains both an −O group and a −OH group, while a yellow color is seen when alizarin has two −OH groups. In TPCM, alizarin changes color according to the pH, which is controlled by *n-*octadecanoic acid at its phase‑transition temperature range of 45–70 °C. In TPCM-Si, the composite was light orange below 46 °C and dark orange above 72 °C. This indicates that alizarin has an −O group and a −OH group at both temperatures, with an interchange of −O groups above 72 °C due to reactions with nanosilicon dioxide. In TPCM-Si-BN, the color changes from dark orange to dark yellow from 46 to 74 °C. This means that alizarin in this composite is in a similar state to that of TPCM. This study produced enhanced thermochromic wood compared to their other works,^31,88,89^ being able to revert to the original color within 60 min below 45 °C and having latent heat of 133.8 J g^–1^ and 163.4 J g^–1^ for TPCM-Si and TPCM-Si-BN, respectively.

**Figure 8:**
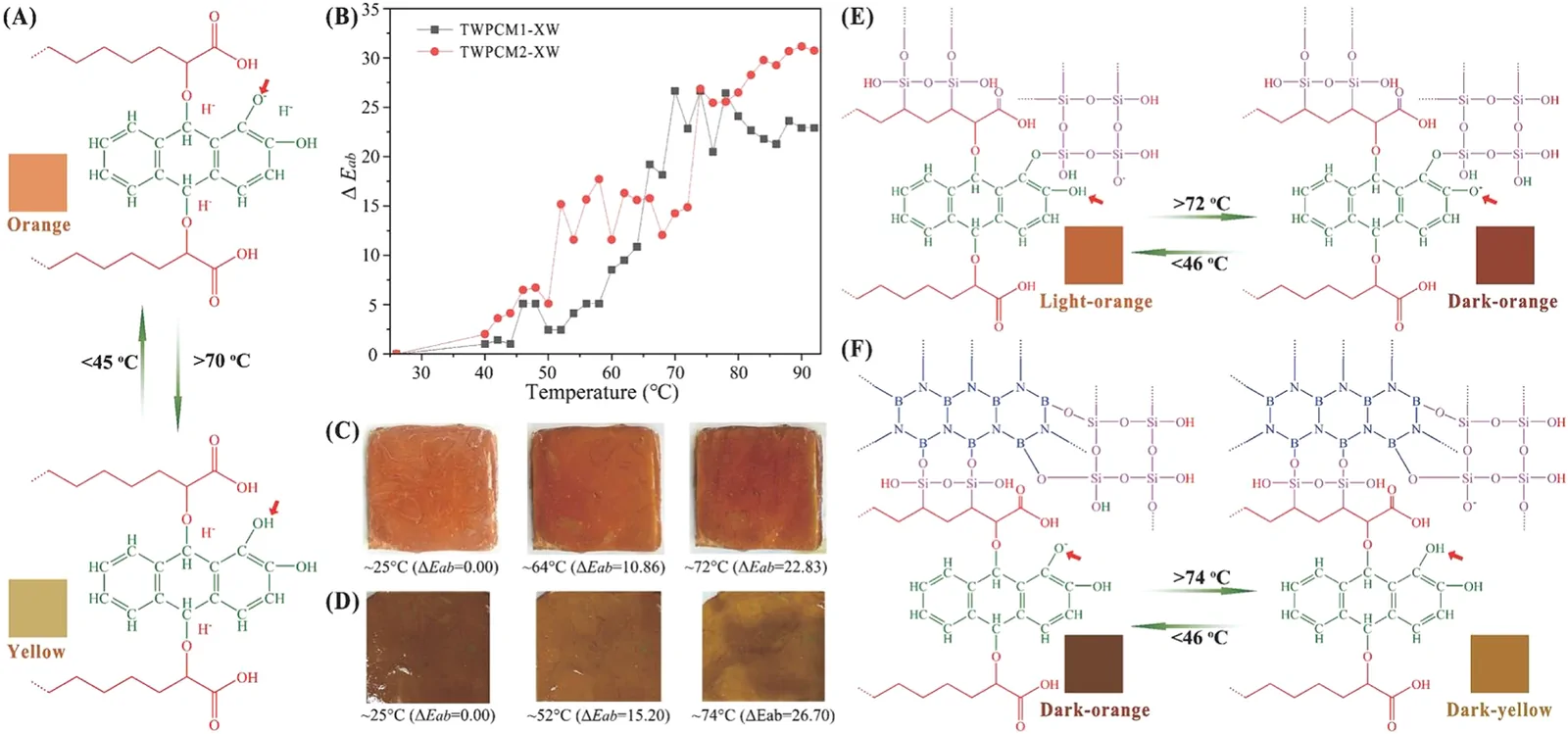
Thermochromic behaviors and mechanisms of TPCM, TPCM-Si, TPCM- Si-BN, TWPCM1-XW, and TWPCM2-XW: (A) Thermochromic mechanism of TPCM; (B) color difference (Δ*E*_*ab*_) curves of TWPCM1-XW and TWPCM2-XW when heated from 26 to 94 °C; (C) thermochromic behavior of TWPCM1-XW; (D) thermochromic behavior of TWPCM2-XW; (E) thermochromic mechanisms of TPCM-Si and TWPCM1-XW; (F) thermochromic mechanisms of TPCM-Si-BN and TWPCM2-XW.^87^ (“Photograph courtesy of ‘Zou et al.,’Copyright 2025”).

In their other study, Zou et al. (2025) fabricated thermochromic poplar and pine wood by impregnation with a ternary compound comprised of natural pigment alizarin, myristyl myristate developer, and 1-hexadecanol cosolvent.^43^ The thermochromic compound-filled wood was then encapsulated with sodium methylsilicate. The wood composite appeared grayish-purple below 52 °C and changed to deep purple above 70 °C, as shown in Figure 9B. Heat-storage capabilities were also realized in the wood composite due to the phase-change properties of myristyl myristate and 1-hexadecanol. As such, the developed thermochromic wood composite was proposed to be used as a thermal warning system and a nonload-bearing overlay material inside buildings.

**Figure 9:**
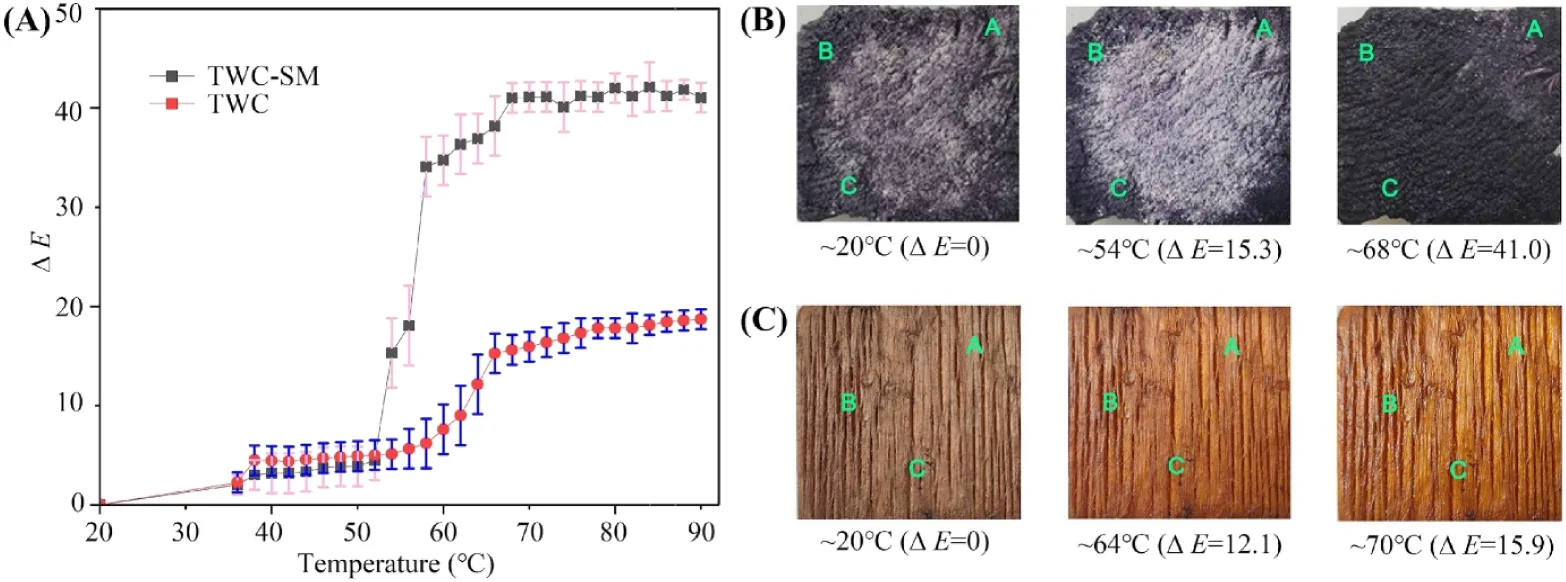
Δ*E* values and surface color images of thermochromic wood composite (TWC) samples and thermochromic wood composite with sodium methylsilicate (TWC-SM) samples at some color-changing temperatures: (A) the Δ*E* values of TWC and TWC-SM at 20–90 °C; (B) the surface color images and Δ*E* values of TWC-2-SM at ∼20 °C, ∼54 °C, and ∼68 °C; (C) the surface color images and Δ*E* values of TWC-2 at ∼20 °C, ∼64 °C, and ∼70 °C. (“Photograph courtesy of ‘Zou et al.,’Copyright 2025”).

Chen et al. (2026) synthesized a negative photochromic spiropyran (NSP) to eliminate the use of potentially toxic components within a conventional DDS thermochromic compound.^90^ Aromatic solvent S1000 was added to inhibit crystallization of NSP, and *n-*octadecane was incorporated as the phase-change core. These three components were then encapsulated in polyurethane shells to create thermochromic microcapsules. The mechanism of negative photochromism in NSP involves thermal coloration (positive thermochromism) and visible-light discoloration, as shown in Figure 10a. A white-to-magenta color change was observed upon heating the microcapsules to 60 °C. The microcapsules recorded melting enthalpy and energy storage efficiency of 180.1 J g^–1^ and 74.9%, respectively, which comes from the incorporation of *n-*octadecane. Thermal cycling stability was consistent for 10 cycles, as shown in Figure 10e. The application of the microcapsules were tested in waste-heat recovery and utilization of wastewater, and the microcapsules were able to provide visual indication for the energy storage process.

**Figure 10:**
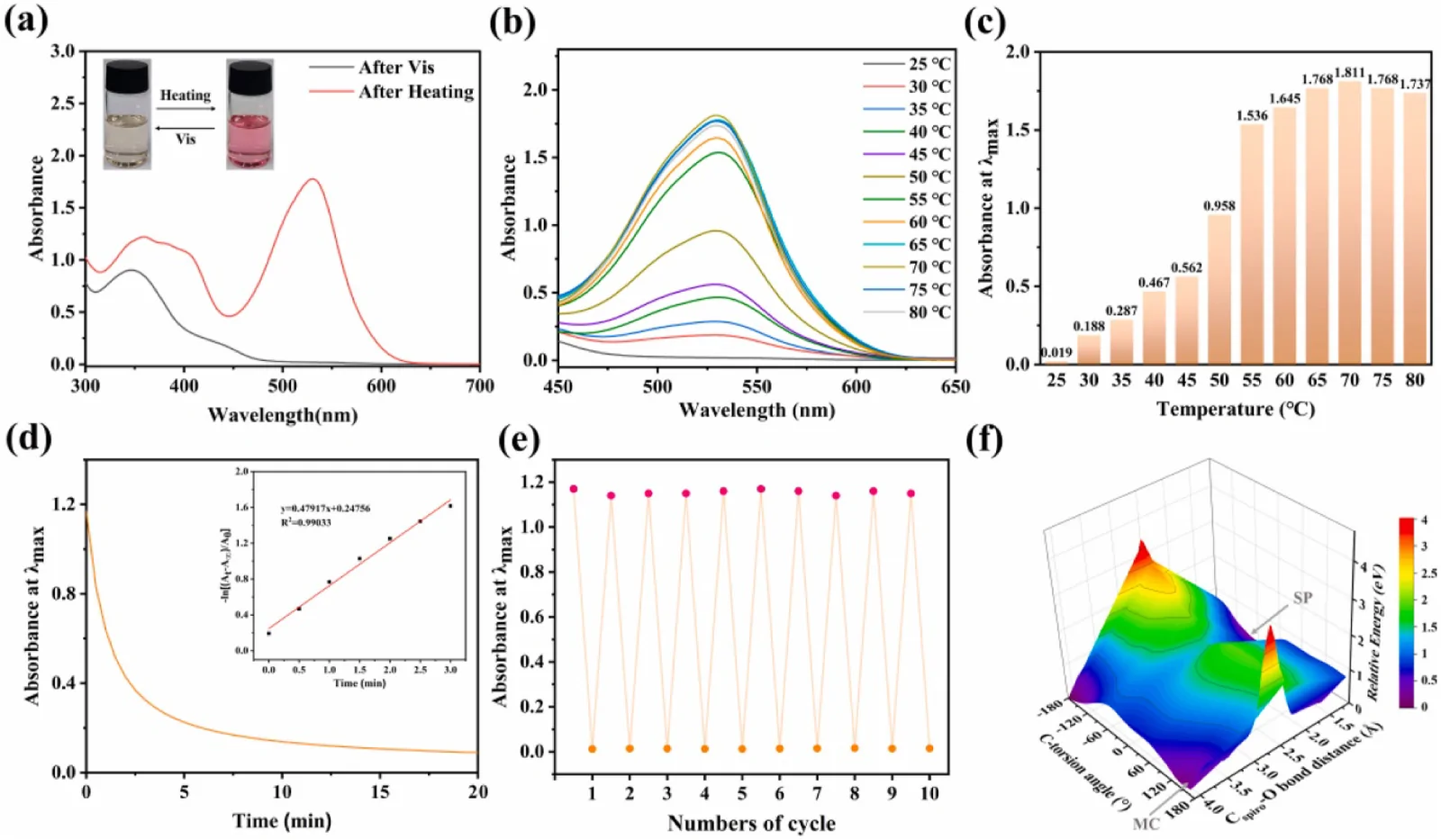
(a) UV–vis absorption spectra of NSP solution; (b) UV–vis absorption spectra of NSP solutions at different temperatures; (c) the absorption intensity of NSP at λ_max_; (d) fading curves and fading kinetic curves at λ_max_ of NSP; (e) fatigue resistance of NSP solution; (f) relaxed two-dimensional scan of the ground-state potential energy surface along the C_spiro_–O distance and the C-torsion angle.^90^ (“Photograph courtesy of ‘Chen et al.,’Copyright 2026”).

### 5.2 Non-Toxic Developers

Zhang et al. (2018) developed thermochromic DDS compounds using bromocresol purple (BCP) as the dye, *n-*octadecanoic acid as the developer, and binary eutectic mixtures as the cosolvent.^21^ Figure 11 shows the mechanism of thermochromism of the compound. Upon heating, the compound turns from dark red to yellow, which is caused by BCP changing reversibly from its cationic form to its closed-ring form between 25 and 60 °C. The constituents of the binary eutectic mixtures used in this study were *n-*dodecanol, *n-*tetradecanol, *n-*hexadecanol, and *n-*octadecanol, and the phase transition temperature of the resultant compounds could be tuned from 13 to 46 °C. This showed that *n-*octadecanoic acid is a viable option as the developer for a low-toxicity DDS compound. The compounds showed stable reversibility for 50 thermal cycles. The proposed application for the compounds was thermal-indicating material.

**Figure 11:**
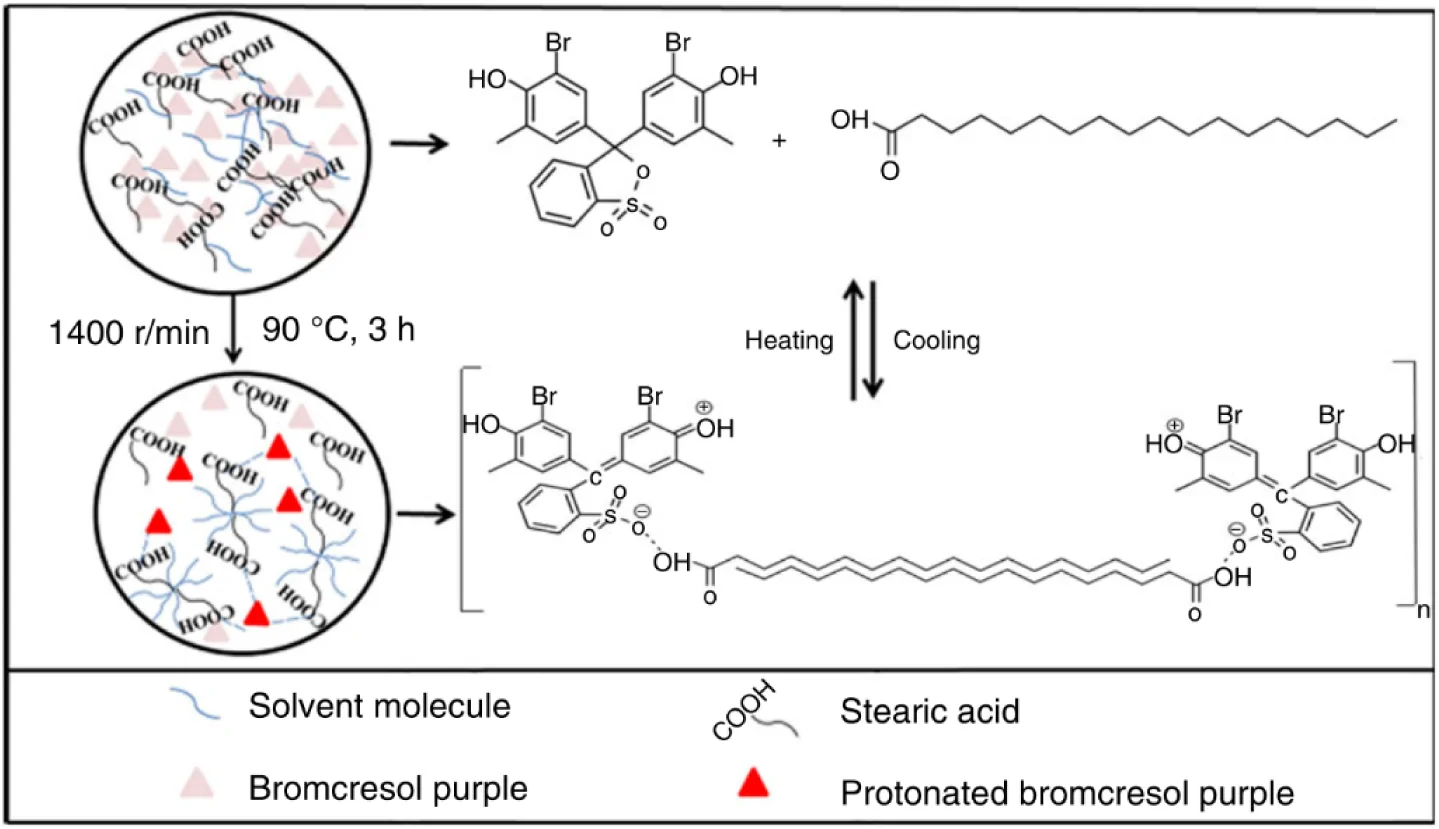
Schematic diagram of the synthesis of a thermochromic colorant using bromocresol purple, *n-*octadecanoic acid, and *n-*octadecanol.^21^ (“Photograph courtesy of “Zhang et al., Copyright 2018”).

Liu et al. (2022) substituted BPA with _L_-ascorbate palmitate (LAP) as the color developer.^12^ Due to the presence of a long alkyl chain in the molecules of LAP, the color changed from colorless to blue, exhibiting positive thermochromism. The schematic representation of this reaction is shown in Figure 12b. The ability to store thermal energy and thermochromic reversibility up to 10 thermal cycles were confirmed, signifying that the new compound functions similarly to a conventional ternary thermochromic compound. The study also revealed that *n-*tetradecanol and *n-*hexadecanol were the most suitable cosolvents, as both imparted thermochromic properties to the compound. On the other hand, the compound with *n-*dodecanol as cosolvent did not exhibit any thermochromic behavior while that with *n-*octadecanol showed only partial thermochromism. This was attributed to weak developer-solvent interaction, which was insufficient to fully separate the dye-developer complex formed. The thermal energy absorption in the CVL/LAP/*n-*tetradecanol compound was found to be similar to that of 10 g water increasing 4.28 °C, making the compound suitable as low-temperature, controllable, color-changing material and heat energy storage.

**Figure 12:**
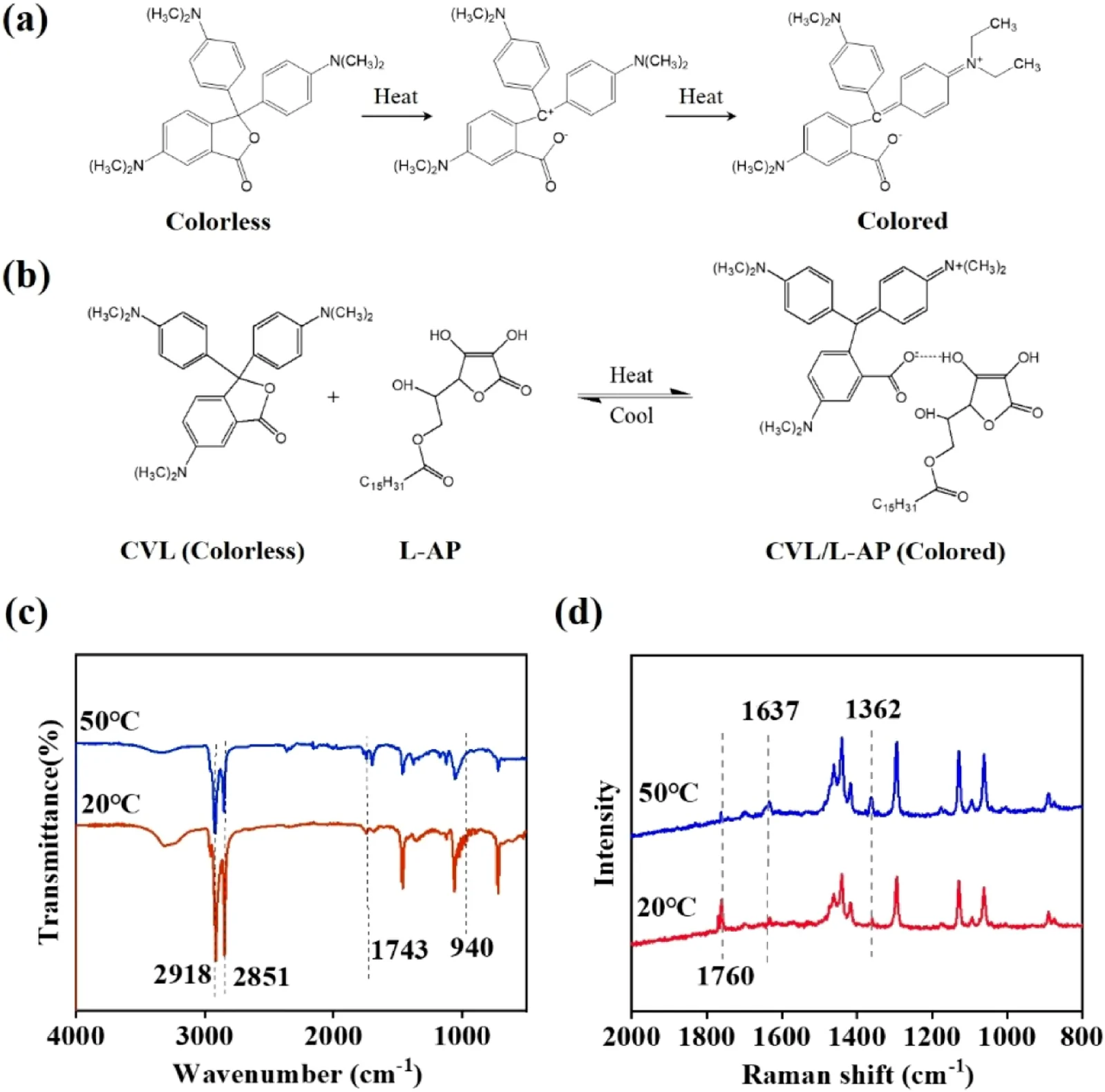
(a) Molecular structures of the ring closed form (colorless), ring-opened carbocationic form, and ring-opened iminium form (colored) of CVL; (b) reaction formula of the colored CVL/LAP formation; the IR (c) and Raman spectra (d) of the thermochromic pigment. (“Photograph courtesy of “Liu et al., Copyright 2023”).

In their next study, Liu et al. (2023) used tea polyphenol instead of BPA as the color developer for thermochromic compounds with several different dyes, which were CVL, DDF, DOF, and 3′,3′-bis­(2′-ethoxy-4′-diethylaminophenyl)-4′-azaphthalide (BEDA).^91^ As shown in Figure 13, the compounds exhibited color changes from a colored state at room temperature to a lighter-colored state, indicating negative thermochromic phenomenon. Upon incorporating the thermochromic dyes into linen, it was found that the elongation at break increased while the tensile strength decreased, which was attributed to the lubricating effect of the dyes, enhancing the slippage between the linen fibers. The thermochromic linen were also added with the ability to store and release heat energy as a result of the solid–liquid phase transition undergone by the solvent of the ternary thermochromic compound.

**Figure 13:**
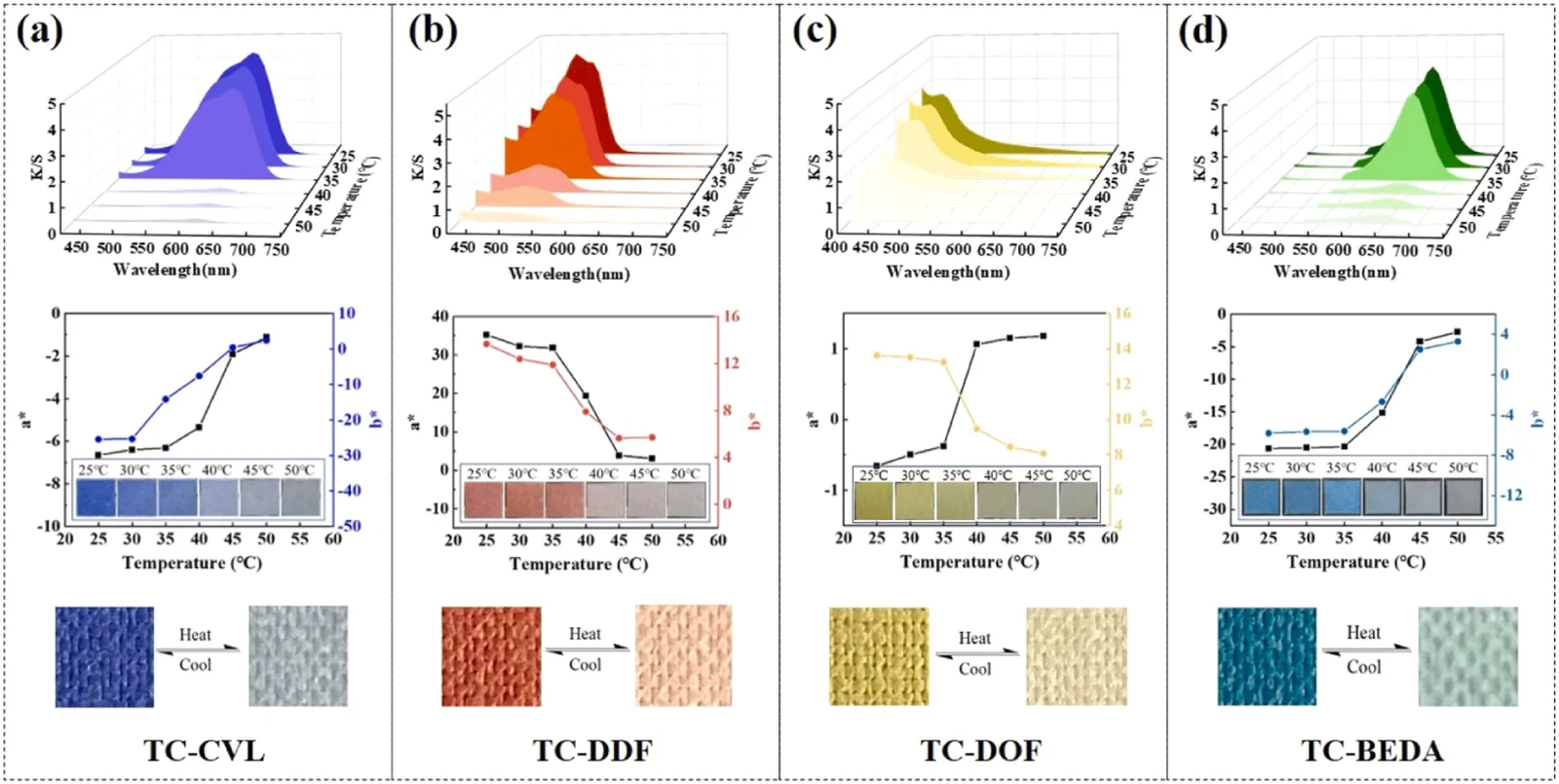
Reversible color-changing performance of thermochromic linen fabric. Temperature-evolved K/S values, temperature-evolved chromatic parameters (a* and b*), and photographs of thermochromic linen fabrics treated with TC-CVL (a), TC-DDF (b), TC-DOF (c), and TC-BEDA (d).(“Photograph courtesy of “Liu et al., Copyright 2023”).

To further their research, Liu et al. (2025) developed flexible thermochromic PDMS films through impregnation with CVL dye, tea polyphenol developer, and *n-*tetradecanol cosolvent.^92^ The films showed color changes between blue and white in the temperature range of 20–50 °C. They found that through adjusting the composition of leuco dyes and cosolvent, the phase-change temperature of the films was tailored to produce multitemperature display, as shown in Figure 14. This shows that the films can be used for multilevel temperature response information transfer.

**Figure 14:**
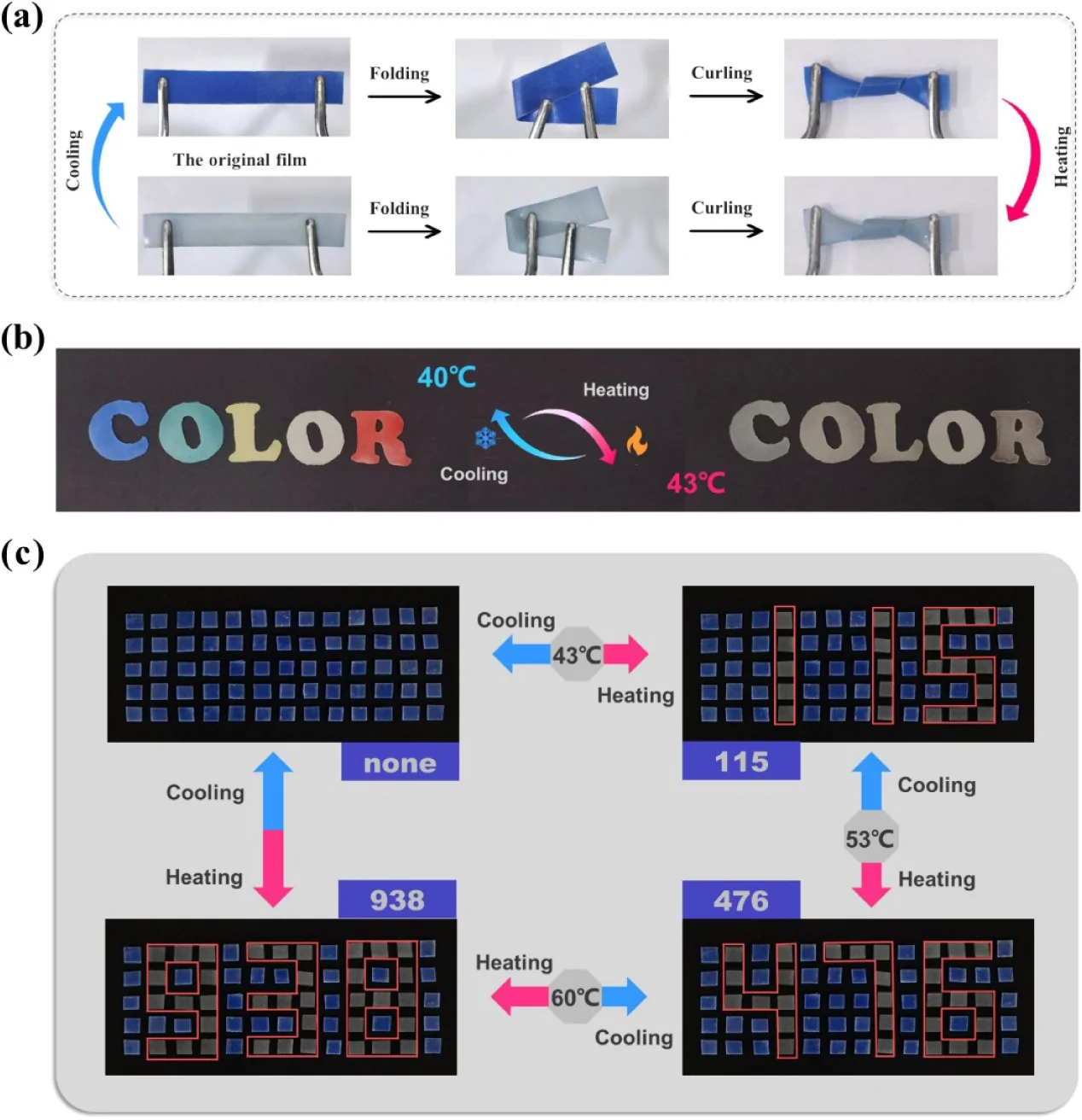
(a) Photographs of thermochromic films with one-sided adhesive properties under original, folding, and curling conditions; (b) multicolor temperature stickers; (c) temperature stickers with different response temperatures.^92^ (“Photograph courtesy of “Liu et al., Copyright 2025”).

Similarly, Dinda et al. (2025) developed a Rhodamine-based thermochromic system using long-chain gallate developers and fatty acid cosolvents.^20^ They studied the effect of the difference in alkyl chain length between cosolvent and developer (Δ*C*) on the thermochromic properties of a compound. The color developers incorporated were octyl gallate, decyl gallate, tetradecyl gallate, hexadecyl gallate, and octadecyl gallate, while the cosolvents used were *n-*decanoic acid, *n-*dodecanoic acid, and *n-*hexadecenoic acid. After coupling the various developers and cosolvents with the dye, compounds with developers having longer alkyl chains than those of cosolvents (Δ*C* < 0) were found to always develop thermochromic properties, while the other samples (Δ*C* ≥ 0) exhibited permanent coloration. As a representative test, the compound with octadecyl gallate and hexadecenoic acid was put through 20 thermal cycles and was found to possess stable reversibility. They also examined the effect of other leuco dyes on the formulation by substituting the lactam-derivative Rhodamine B dye with lactone and fluoran dyes. The compounds with the other dyes, 2′-(2-chloroanilino)-6′-(dibutylamino)­fluoran (FLU2) to name one, showed colorless-colored and white-colored thermochromism at 32 °C in octadecyl gallate/*n-*decanoic acid (ΔC = −6) and octyl gallate/*n-*decanoic acid (Δ*C* = +2), respectively. The proposed applications of the compounds include sensors and invisible security inks.

Zhao et al. (2026) developed a three-stage thermochromic compound with thermochromic green dye 2′-anilino-6′-(phenylamino)-diethylaminospiro­[phthalide-3,9′-[9H]­xanthene] (TCG), DG, and decyl decanoate.^93^ Decyl decanoate was incorporated to function as a cosolvent and developer modifier. The compound changes from green to white around 0 °C and from white to green around 40 °C. The two-step color change observed was attributed to possible phenomena as follows: below the lower phase transition temperature (*T_L_*), the crystallization of decyl decanoate caused DG to release protons; between *T_L_* and the higher phase transition temperature (*T_H_*), the self-assembly of DG resulted in it undergoing phase separation from the solvent; and above *T_H_*, DG molecules are free to interact with dye molecules, thus forming the green compound. The schematic representing these changes is shown in Figure 15. The thermochromic response time was found to be less than 60 s, and the reversibility was stable for over 500 thermal cycles. The proposed applications are commercial temperature monitoring and anticounterfeiting.

**Figure 15:**
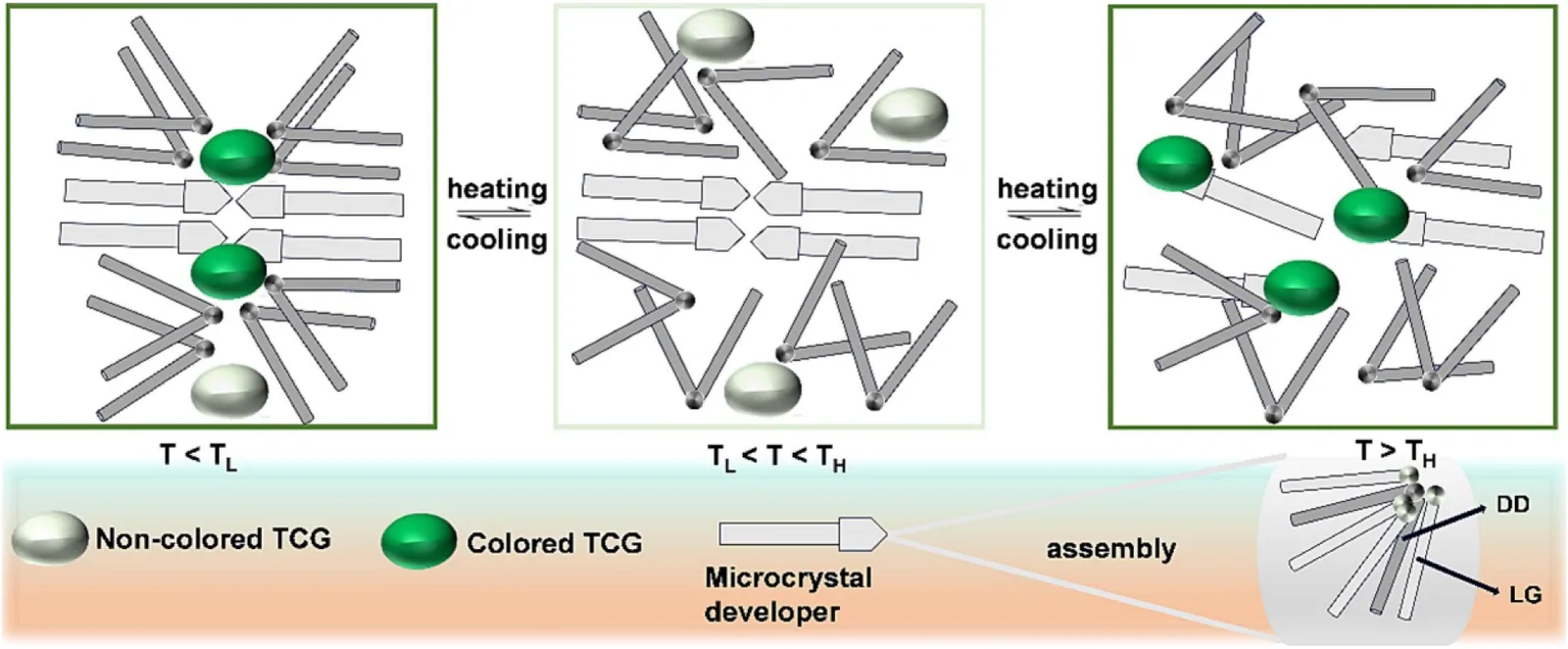
Schematic view of possible dynamic structures for triple-stage thermochromic material.^93^ (“Photograph courtesy of “Zhao et al., Copyright 2026”).

Jie et al. (2026) developed flame-resistant thermochromic wood composites using the DDS compound BCP/*n-*decanoic acid/*n-*tetradecanoic acid.^94^ The flame-retardant MF coating applied to the thermochromic wood was composed of chitosan and phytic acid, which are ingredients that can be obtained from sea animals and plants, respectively. In the thermochromic compound, proton transfer was theorized to happen from *n-*tetradecanoic acid to BCP, causing a ring-opening reaction and thus changing the color from light green to yellow between 16 and 40 °C. The coated thermochromic wood composite showed excellent flame-retardant properties, which were attributed to phytic acid promoting the forming of a robust carbon layer from chitosan on the surface during combustion, paired with the release of inert gases from MF, catalyzed by phytic acid. Both of these phenomena provide a synergistic effect that blocks heat transfer and lowers the oxygen concentration, enhancing the flame-resistance.

### 5.3 Alternative Mechanisms

Liu et al. (2019) tested various thermochromic binary systems with different dyes and solvents.^17^ The color change is triggered by the change in pH caused by the melting or crystallization of the solvent, changing the π–π stacking in the dye, known as aggregachromism. First, they mixed *n-*dodecanoic acid solvent with sulphonephthalein dyes chlorophenol red (CPR), bromothymol blue, bromocresol purple, bromocresol green, bromophenol blue, and thymol blue, as well as charged triarylmethane dyes methyl green and crystal violet, leuco dye CVL, and azo dye dimethyl yellow. Upon evaluating the color changes between 25 and 70 °C, CPR was found to be the most suitable dye due to the sample having the highest Δ*E* value. In the next phase, CPR was mixed with hexadecenoic acid, methyl hexadecanoate, and *n-*hexadecanol to test the functionality of each type of solvent. All solvent types were found to impart thermochromic properties with temperature change. Following that, CPR was mixed with two different concentrations of *n-*dodecanol, *n-*tetradecanol and *n-*hexadecanol to test the effect of solubility of the dye in the solvent matrix on thermochromism. They discovered that a decrease in solubility promoted phase separation, thus imparting thermochromism to the mixture.

Liu et al. (2020) followed up the previous study by developing a thermochromic system with CPR and water.^95^ The optimal concentration of CPR in the aqueous phase was found to be 10^–3^ M, showing the highest Δ*E* of 44.6 between frozen and melted forms. The observed color changed reversibly from orange to purple between −196 and 25 °C. Encapsulation of the thermochromic compound with silicone was successful, allowing the incorporation of the microcapsules into ink and PDMS films. The films were fully reversible throughout the 30 thermal cycles between −80 and 25 °C.

Zou et al. (2021) developed thermochromic poplar wood using methyl red dye and tetradecyl tetradecanoate developer.^31^ The thermochromic wood sample was then encapsulated with propylene wax to prevent leaking of thermochromic compound from the wood. The melting peak temperature decreased when the thermochromic compound was incorporated into the poplar wood, which was attributed to the covalent bond between the compound and wood fibers. Color change from light red to dark red was observed in the temperature range 38–46 °C and is reversible after 14 h below 38 °C. The proposed thermochromic mechanism was proton transfer between methyl red and tetradecyl tetradecanoate. Below the melting temperature, the developer is in its solid form and does not interact with methyl red, leaving it in its acidic form. As the temperature increases, the developer melts and methyl red undergoes deprotonation and becomes its alkaline form, thus contributing to the mentioned color change in the thermochromic compound.

In a follow-up study, Zou et al. (2022) fabricated red thermochromic poplar wood consisting of methyl red dye and *n-*decanoic acid solvent.^88^ Color change was observed between light red and dark red after 3 min of heating, with the phase-change temperature range being 24–40 °C. The color reversion can be achieved after 4 h below 22 °C. The thermochromic mechanism is similar to the previous study, which is the change in pH controlled by the melting and crystallization of *n-*decanoic acid, as the solvent/developer.

Next, Zou et al. (2024) further refined the thermochromic poplar wood composite by examining the effect of immersion time in silica sol and dye/solvent ratio on the properties of the composite.^89^ The thermochromic compound used was methyl red/tetradecyl tetradecanoate. They found that the immersion time did not affect the color of the composite, and a moderate dye/solvent ratio of 3:1000 was sufficient to induce very obvious color changes. In general, light purple to dark purple color change was observed in the wood composite at the temperature range of 30–48 °C, and the thermochromic mechanism was the deprotonation/protonation of methyl red by the solvent.

Zhang et al. (2026) developed an irreversible fabric-based composite with three layers: the top layer was PEG, the middle layer was polyester fabric, and the bottom layer was PMMA photonic crystals.^96^ The thermochromic mechanism is triggered when the PEG layer is melted by increasing temperature, migrating downward through the fabric and into the PMMA photonic crystal layer. This fills the interstitial spaces between photonic crystal nanospheres, altering decreasing the contrast in refractive index within, discoloring the photonic crystal layer. This phenomenon is not reversible, as PEG solidifies upon cooling. Instead, the color of the fabric is shown after the thermochromic color change. The phase transition temperature and color can be finetuned by manipulating the molecular weight of PEG and the PEG/water mass ratio. The composites fabricated can undergo color changes at 5 °C, 10 °C, 20 °C, and 50 °C when PEG with molecular weight of 600, 1000, 1500, and 2000 were incorporated, respectively. These composites can record the thermal history of products in cold-chain logistics.

While the thermochromic compounds mentioned in this subsection do not rely on a DDS system, these studies give a glimpse into the mechanism of color change, which is based on a pH change due to the melting of the solvent. These studies provide some insight into alternative designs in which thermochromic color switching can be triggered. This mechanism could be applied in fabricating thermochromic compounds with natural pigments as the dye.

### 5.4 Cytotoxicity Tests

A few studies mentioned in the previous sections have tested the cytotoxicity of the samples on various human cell samples. Liu et al. (2019) assessed the toxicity of CPR on NIH-3T3 (noncancerous fibroblasts) cells and found that the toxicity was relatively low in comparison to that of cyanidin chloride and rhodamine B.^17^ Hence, the authors proposed commercial food and healthcare packaging applications for binary thermochromic mixtures. Liu et al. (2020) tested microcapsules and films with CPR/water thermochromic mixture, which were found to be nontoxic on NIH-3T3 cells, thus suitable as temperature sensors, coatings, and packaging of frozen products.^95^ Feng et al. (2026) tested methylcellulose-shelled microcapsules with varying concentrations of JYGreen-1, 2-naphthol as the developer, and methyl dodecanoate as the cosolvent with human umbilical vein endothelial cells (HUVEC) for 24 h.^90^ The HUVECs showed high viability, indicating that the microcapsules had good biosafety. Hence, the authors recommend using the compounds for smart fabrics in cold-chain delivery or temperature indication in cold weather. It is worth noting that encapsulation kept the cytotoxicity low upon fabrication, despite the compounds containing 2-naphthol, which is harmful to human health.^79^ However, a concern for this is the long-term safety of the microcapsules, as the capsule shell may degrade over time, allowing the toxic components to leak out and affect the health of users and the environment.

### 5.5 Design Challenges and Suggestions for Future Research

Table 7 summarizes the advantages, limitations, similarities, and proposed applications of conventional and nontoxic DDS compounds. As seen from the examples discussed in the above sections, there is potential to produce environmentally friendly and nontoxic thermochromic polymers by substituting synthetic or harmful ingredients with natural or safer alternatives. This can be done through understanding the thermochromic mechanisms that govern color changes in the desired temperature range. However, there are still some challenges that must be overcome in order to realize this potential.

**Table 7:** Comparison between Non-Toxic and Conventional DDS Compounds

	Conventional DDS compounds	Nontoxic DDS compounds
Dye	Synthetic fluoran, azo dyes	Plant-based dyes such as anthocyanins
Developer	BPA, DG	DG, plant-based developers (LAP, tea polyphenols)
Cosolvent	Fatty acids, alcohols, *n*-alkanes, alkyl esters, alkyl benzoates, binary eutectic mixtures	Fatty acids, alcohols, binary eutectic mixtures
Advantages	• Well-researched with established database.	• Renewable from natural resources.
	• Materials easily available on the market.	• Low- or nontoxic to users and the environment.
	• Incorporation methods provide good versatility in applications.	
	• Wide range of colors due to the many different types of synthetic dyes available.	
Limitations	• Contains toxic ingredients such as CVL and BPA.	• Currently lacks commercialized extraction methods for the raw materials.
	• Lack sustainability due to usage of synthetic components.	• Poor thermal stability due to the incorporation of natural pigments.
		• Color transition is currently limited to being irreversible or from positive thermochromism only.
Similarities	• Phase transition temperature can be controlled by manipulating the cosolvent composition.	
	• Thermal cycling stability comparable for both types due to the similar cosolvent used.	
Proposed applications	• Architecture	• Encryption
	• Encryption	• Food packaging
	• Food packaging	• Healthcare
	• Furniture	• Textiles
	• Healthcare	• Thermal management
	• Leisure	
	• Textiles	
	• Thermal management	

One difficulty is the compatibility of the alternative components, mainly the dye or developer, with the other components. For instance, the developers used in previous studies have either linear (e.g., fatty acids, alkyl esters) or simple structures (e.g., BPA),^13,21^ which do not hinder interaction with the dye and cosolvent. In naturally sourced ingredients, the molecular structure (e.g., anthocyanins, tea polyphenols) may be more complex,^35,91^ and this may restrict access to the functional groups that alter the state of the dye or cause poor compatibility with the cosolvent due to differences in hydrophilicity. This may give rise to poor interaction between the dye and developer or solvent, which may affect the phase separation that produces thermochromic color change, as seen in a previous study.^17^ Hence, future research can continue to experiment with different plant-based pigments and developers. New alternative components can be inserted into established thermochromic systems to observe changes in thermochromism or identify possible hurdles to achieve thermochromism. Each effort to understand the interaction between the new ingredient and the rest of the system is a valuable addition to the database of thermochromic polymers.

Although anthocyanins are biodegradable, their use poses some issues that are yet to be resolved.

Since natural pigments were found to exhibit only color changes in response to hydrogen bonding and pH change, there is more room for research into suitable mechanisms to trigger a thermochromic change in natural dye-based DDS systems. As of now, natural pigments have been found to be suitable for use in systems that utilize the migration of the solvent across layers of polymer to trigger thermochromic changes.^35,86^ For natural pigment derivatives such as cyanidin chloride, interaction with PLA molecules caused a thermochromic color change, but the reversibility was not discovered.^84^ These permanent color changes could be useful in a single-use temperature sensor to provide the thermal history of the product, that it has been removed from the appropriate storage temperature for a period of time.^96^ However, it prevents the sensor from being reusable and thus would require replacement, which can be a concern toward higher cost and lower sustainability. Therefore, a balance needs to be achieved between fabricating renewable, natural-pigment-based DDS compounds and reversible thermochromism, depending on the desired application and cost or availability of materials.

Because of poor thermal stability, anthocyanins may be difficult to pair with other components that have high melting points, as that may cause degradation and ultimately destroy the functionality of the thermochromic compound upon mixing or after a few uses. One method of improving thermal stability of thermochromic compounds is encapsulation.^48^ The capsule shell also confines the compound within, preventing it from leaching from the polymer matrix and increasing thermal conductivity and thermochromic response.^49^ Besides encapsulation, the addition of antioxidants may help preserve the color-changing properties of natural pigments at an elevated temperature. Future studies can examine the effect of antioxidants on natural pigment-based thermochromic systems. However, one consideration would be whether the antioxidant species interferes with the thermochromic mechanism. If the thermal stability of the pigments can be improved, it may be possible to use it in a DDS compound with a high-melting-point cosolvent, which extends the functional temperature range of such compounds.

## 6 Conclusion

Research in the field of thermochromic polymers has seen much progress, thanks to the increasing interest in smart materials. These materials rely on the equilibrium between dye-developer and developer–solvent interactions to work. When the dye–developer interaction is strong, the color of the leuco dye is visible; when the developer–solvent interaction is favored, the leuco dye loses its color. Thermochromic DDS compounds provide a promising future for thermochromic polymers, granting great tunability in terms of dye color, direction of color change, and phase-change temperature. Until now, further research is still required to develop more nontoxic materials that possess thermochromic capabilities, as there is still plenty of room for exploration regarding the use of natural resources, such as natural pigments and other bioactive substances. Through the discovery of new and safer thermochromic polymers, real-time temperature monitors can be applied in fields such as the storage and transportation of food and pharmaceutical goods.
